# A new branch of mammalian vitamin B_6_ metabolism: AKR1C‐mediated conversion of pyridoxal to pyridoxine and 4‐pyridoxolactone

**DOI:** 10.1111/febs.70471

**Published:** 2026-02-25

**Authors:** Nayu Kito, Yasuyuki Kitaura, Kyoka Iino, Kazuya Toriumi, Hyunah Noh, Naoya Ogawa, Makoto Arai, Hisashi Hemmi, Tomokazu Ito

**Affiliations:** ^1^ Department of Applied Biosciences, Graduate School of Bioagricultural Sciences Nagoya University Aichi Japan; ^2^ Department of Food and Nutritional Sciences, College of Bioscience and Biotechnology Chubu University Aichi Japan; ^3^ Schizophrenia Research Project, Department of Clinical Medical Sciences, Tokyo Metropolitan Institute of Medical Science Japan

**Keywords:** AKR1C, aldo‐keto reductase, pyridoxal, pyridoxal reductase, vitamin B_6_

## Abstract

Pyridoxal 5′‐phosphate (PLP), the coenzyme form of vitamin B_6_, is indispensable for diverse metabolic processes, especially amino acid metabolism. In mammals, PLP is primarily synthesized via a salvage pathway involving pyridoxal kinase (PLK), pyridoxine/pyridoxamine 5′‐phosphate oxidase (PNPO), and pyridoxal phosphate phosphatase (PLPP). However, recent evidence suggests the presence of additional, yet unidentified, enzymatic contributors to this pathway. Here, we identify aldo‐keto reductase family 1 member C (AKR1C) isozymes as previously unrecognized enzymes involved in vitamin B_6_ metabolism. We demonstrate that AKR1Cs catalyze two novel reactions: an NADPH‐dependent pyridoxal reductase (PLR) activity that converts pyridoxal (PL) to pyridoxine (PN), and an NADP^+^‐dependent pyridoxal dehydrogenase (PLD) activity that oxidizes PL to 4‐pyridoxolactone (4‐PLA). Both reactions occur under physiological conditions and significantly impact intracellular vitamin B_6_ vitamer profiles. Moreover, we show that elevated PL levels suppress AKR1C activities toward non‐B_6_ substrates, indicating reciprocal cross‐talk between vitamin B_6_ metabolism and other AKR1C‐dependent metabolic processes. This study expands the current framework of mammalian vitamin B_6_ metabolism, highlighting AKR1Cs as metabolic hubs with broad regulatory implications.

## Graphical abstract

Pyridoxal 5'‐phosphate (PLP), the coenzyme form of vitamin B_6_, is essential for amino acid metabolism. In mammals, PLP homeostasis relies on salvage enzymes, yet key metabolic branches remain undefined. We identify AKR1C isozymes as previously undescribed contributors that convert pyridoxal into pyridoxine or 4‐pyridoxolactone through reductase and dehydrogenase activities. These reactions may reshape intracellular vitamin B_6_ vitamer balance and attenuate AKR1C activities towards non‐B_6_ substrates. Our findings establish AKR1C isozymes as metabolic nodes linking vitamin B_6_ availability with broader cellular metabolic regulation, highlighting AKR1Cs as metabolic hubs with broad regulatory implications.

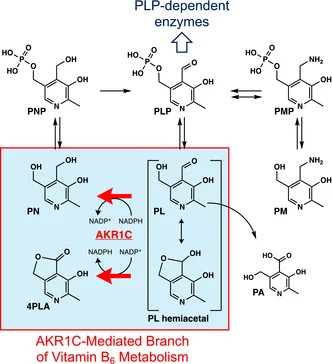

**Abbreviations**
AKR: aldo‐keto reductase
PL: pyridoxal
PLD: pyridoxal dehydrogenase
PLP: pyridoxal 5′‐phosphate
PLR: pyridoxal reductase
PM: pyridoxamine
PMP: pyridoxamine 5′‐phosphate
PN: pyridoxine
PNP: pyridoxine 5′‐phosphate

## Introduction

**Fig. 1:**
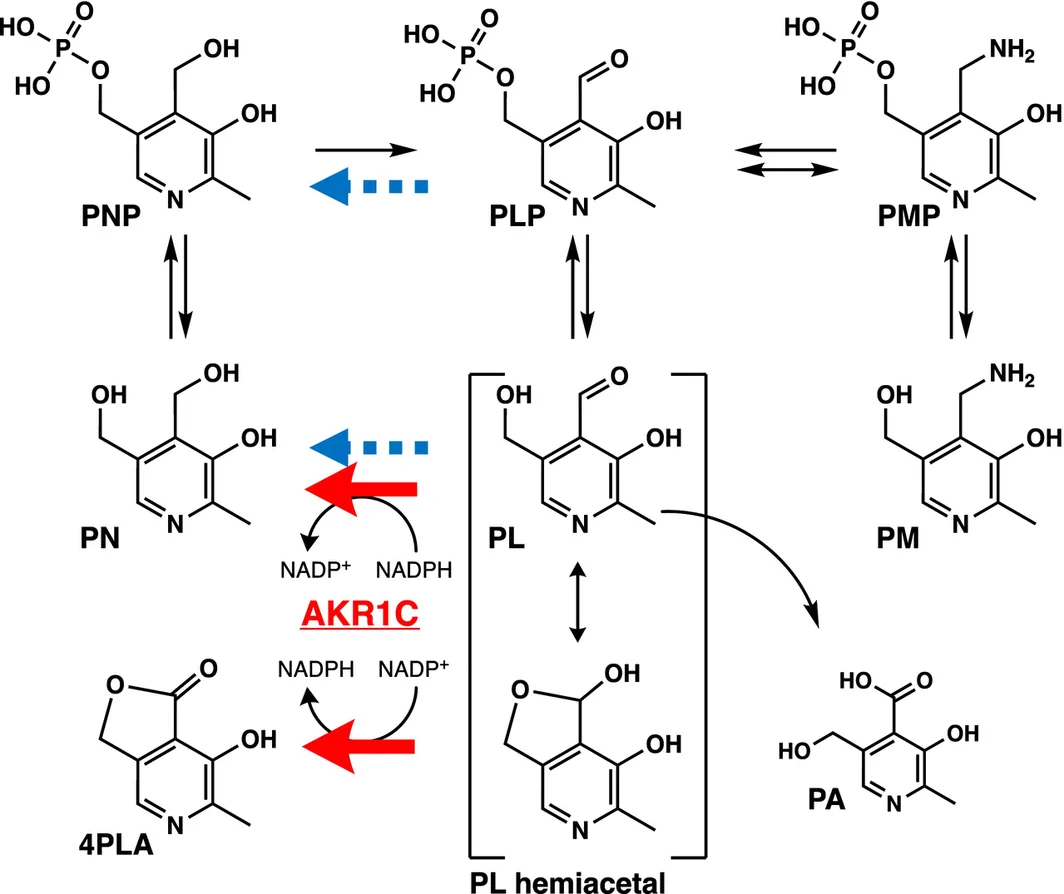
Salvage pathway of vitamin B_6_ in mammals. Vitamin B_6_ metabolism in mammals involves pyridoxal 5′‐phosphate (PLP) synthesis via the salvage pathway, traditionally attributed to pyridoxal kinase (PLK), pyridoxine 5′‐phosphate oxidase (PNPO), and pyridoxal phosphatase (PLPP) (black arrows), and catabolism by aldehyde oxidase (AOX), which converts pyridoxal (PL) to pyridoxic acid (PA). Reports of pyridoxine (PN) and pyridoxine 5′‐phosphate (PNP) accumulation following PL or PLP administration indicate additional enzymatic steps (blue dashed arrows). This study reveals that AKR1C isozymes are novel contributors to vitamin B_6_ metabolism and introduce a branched route (red arrows), in which AKR1C enzymes catalyze the NADPH‐dependent reduction of PL to PN via pyridoxal reductase (PLR) activity and the NADP^+^‐dependent oxidation of PL to 4‐pyridoxolactone (4‐PLA) via pyridoxal dehydrogenase (PLD) activity.

Vitamin B_6_ is the generic name of six interconvertible vitamers: pyridoxal (PL), pyridoxine (PN), pyridoxamine (PM), and their respective phosphorylated forms: pyridoxal 5′‐phosphate (PLP), pyridoxine 5′‐phosphate (PNP), and pyridoxamine 5′‐phosphate (PMP) (Fig. 1). Pyridoxic acid (PA), a catabolic product of PL, is excreted in mammalian urine [1]. Among these vitamers, PLP serves as an essential cofactor for various enzymes involved in amino acids, keto acids, and amine metabolisms, including neurotransmitter biosynthesis and several other essential biochemical processes [2, 3, 4].

Most microorganisms and plants can synthesize PLP *de novo*, via either the deoxyxylulose 5‐phosphate (DXP)‐dependent or DXP‐independent pathways [5, 6]. In contrast, animals lack a *de novo* biosynthetic pathway and rely on dietary sources and/or intestinal bacteria. Animals are capable of utilizing all B_6_ vitamers as precursors for PLP. Phosphorylated forms are absorbed after dephosphorylation by tissue‐nonspecific alkaline phosphatase [7, 8] and intracellular conversion to PLP occurs through the salvage pathway (Fig. 1) [9].

Currently, three enzymes are identified for the salvage process in mammals; pyridoxal kinase (PLK), pyridoxine/pyridoxamine 5′‐phosphate oxidase (PNPO), and PLP phosphatase (PLPP) (Fig. 1) [9]. PLK catalyzes the ATP‐dependent phosphorylation of the 5′ OH group of PL, PM, and PN to their respective phosphorylated forms [10, 11]. PNPO, an FMN‐dependent enzyme, oxidizes PNP and PMP to PLP [12, 13]. PLPP dephosphorylates PLP, PNP, and PMP to corresponding dephosphorylated vitamers in the order of efficiency PLP > PNP > PMP in the cells [14]. Complex mechanism of these enzymes and the absorption and distribution maintain cellular PLP homeostasis.

Defects in salvage enzymes cause PLP deficiency, leading to several pathologies, including B_6_‐dependent epilepsy and polyneuropathy [15, 16, 17]. Moreover, accumulating evidence highlighted the importance of vitamer balance for metabolic and physiological integrity. For instance, altered B_6_ status has been reported in patients with schizophrenia, as summarized in meta‐analyses and umbrella reviews, suggesting a possible involvement of B_6_ metabolism in disease pathophysiology [18, 19, 20]. Historically, vitamin B_6_ supplementation, including PN or PM, has been explored as a therapeutic approach [21, 22, 23]. In plants, excess PMP disrupts the nitrate‐to‐ammonia conversion [24, 25]. In bacteria, PNP accumulation perturbs multiple metabolic pathways, such as Ile‐Val and one‐carbon metabolism, by competing with PLP‐dependent enzymes [26, 27, 28]. A comprehensive understanding of vitamin B_6_ metabolism is critical for elucidating its biological roles and developing therapeutic strategies for vitamer imbalances.

Recent evidence suggests that an additional enzyme(s) may play a role in the B_6_ vitamer metabolism in animals. Patients with vitamin B_6_‐dependent epilepsy treated with high‐dose PLP exhibit elevated PN and other B_6_ vitamers in cerebrospinal fluid and serum [29, 30, 31]. Similarly, PNPO‐deficient (*pnpo* ^*−/−*^) zebrafish supplemented with PLP accumulate PN and PNP [32]. These observations imply the existence of a PL to PN or PLP to PNP conversion pathway in these organisms (Fig. 1, broken blue lines). Supporting this, Ramos *et al*. provided direct evidence for the pathway(s) in mammals by demonstrating that mammalian cell lines (HEK293, Neuro2a, Caco2, and HepG2) supplemented with PL secrete PN in a time‐ and dose‐dependent manner [31]. However, the identity of the enzyme(s) mediating these conversions remains unknown.

To date, the enzyme that catalyzes the reduction of PL to PN, pyridoxal reductase (PLR), has been identified in *Saccharomyces cerevisiae*, *Schizosaccharomyces pombe*, *Arabidopsis thaliana*, and *Escherichia coli* [33, 34, 35, 36]. In these organisms, PLR functions as an additional salvage enzyme, representing a fourth enzymatic component that operates alongside PLK, PNPO, and PLPP in the vitamin B_6_ salvage pathway [33, 35, 36]. All characterized PLRs belong to the Aldo‐Keto Reductase (AKR) superfamily and catalyze NADPH‐dependent redox reactions [33, 35, 36, 37]. Ramos *et al*. investigated some human AKR proteins—including the auxiliary β2 subunit of voltage‐gated potassium channels (AKR6A5), aldose reductase (AKR1B1), and aldose reductase‐like protein (AKR1B10)—as PLR candidates. However, pharmacological inhibition and knockout of these enzymes did not significantly affect PL‐to‐PN conversion efficiency in HepG2 cells, suggesting additional or redundant enzymes exist in mammals [31].

Here, we report previously unrecognized branched pathways of PL metabolism in mammals: conversion of PL to PN and to 4‐pyridoxolactone (4‐PLA). The AKR1C isozymes are identified as bifunctional enzymes in PL metabolism that exhibit NADPH‐dependent PL to PN reduction activity and NADP^+^‐dependent PL to 4‐PLA oxidation activity. Our findings reveal AKR1C enzymes as key regulatory nodes in vitamin B_6_ homeostasis in mammals, which uncovers a previously uncharacterized layer of metabolic control.

## Results

### Occurrence of conversion pathway of PL to PN in mice

**Fig. 2:**
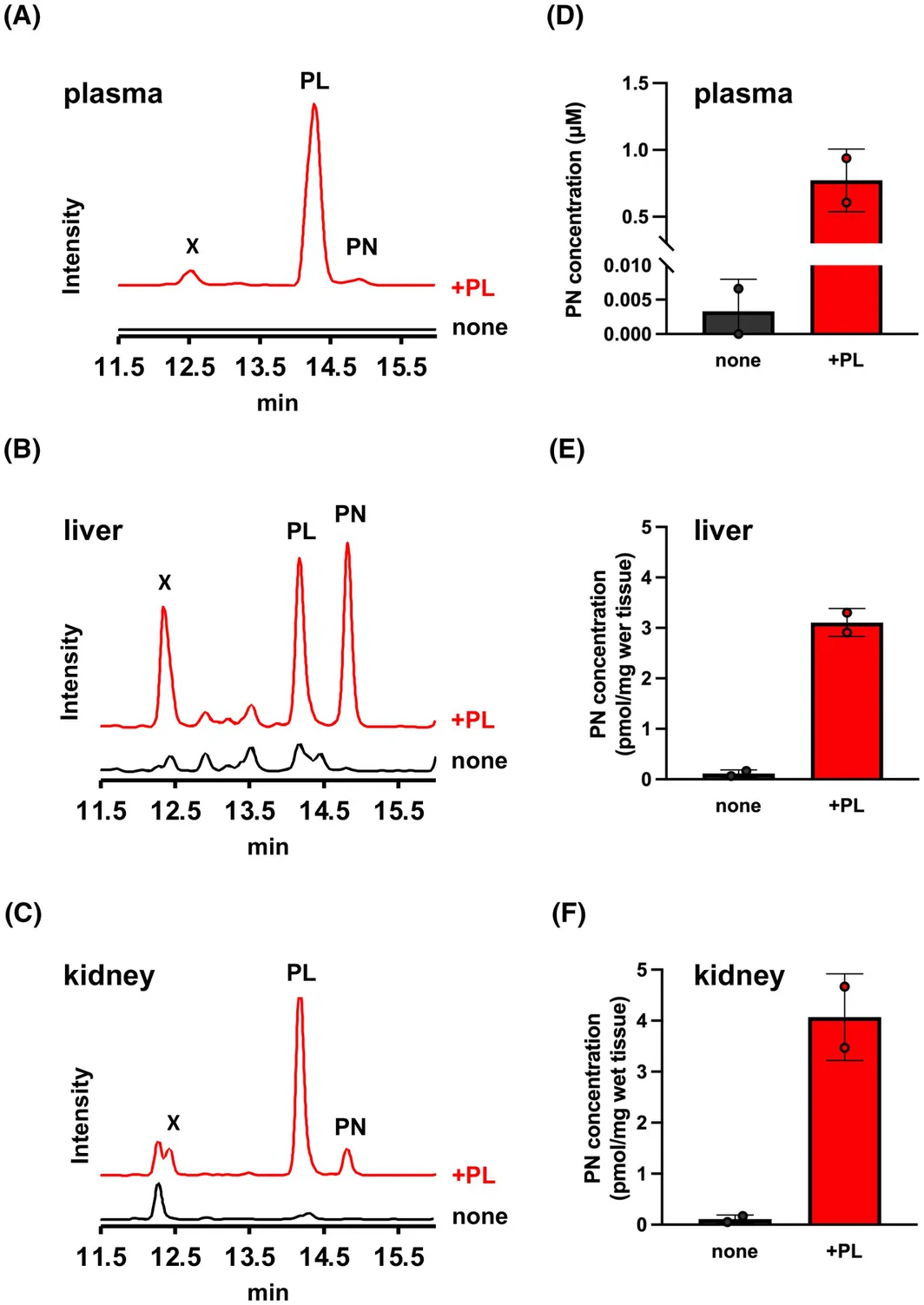
Effect of oral pyridoxal (PL) administration on vitamin B_6_ composition in mice. Mice were administered a single oral dose of PL (0.5 mL of 10 mg·mL^−1^ PL in water) or an equal volume of water as a control. After 1.5 h, blood and tissues were collected, and plasma, liver, and kidney samples were prepared for vitamin B_6_ vitamer analysis. The B_6_ vitamers were separated and quantified by high‐performance liquid chromatography (HPLC) using authentic standards for peak identification. (A–C) Representative chromatograms of vitamin B_6_ vitamers in the plasma (A), liver (B), and kidney (C) of PL‐treated mice (red traces, +PL) and water‐treated control mice (black traces, none) from *n* = 2 independent experiments, all of which showed comparable elution profiles. (D–F) Quantification of pyridoxine (PN) levels in plasma (D), liver (E), and kidney (F). PN levels were significantly elevated in all examined tissues of PL‐treated mice compared with controls, indicating *in vivo* conversion of administered PL to PN. Values represent the mean from two independent experiments (*n* = 2 mice).

The presence of a PL‐to‐PN conversion pathway has been suggested only in a limited number of species, such as humans and zebrafish [29, 30, 31, 32]. To determine whether this pathway also occurs in mice, we administered PL or water to mice and analyzed vitamin B_6_ vitamers in plasma, liver, and kidney. Upon PL supplementation, plasma PN concentration was significantly increased (more than 80 times) to reach 0.8 μm (Fig. 2A,D). Similarly, in the liver and kidney, PN concentrations were markedly elevated (0.1 → 3.1 pmol·mg^−1^ in liver, 0.1 → 4.1 pmol·mg^−1^ in kidney, Fig. 2B,C,E,F). These results confirmed the presence of a PL‐to‐PN metabolic pathway in mice.

**Fig. 3:**
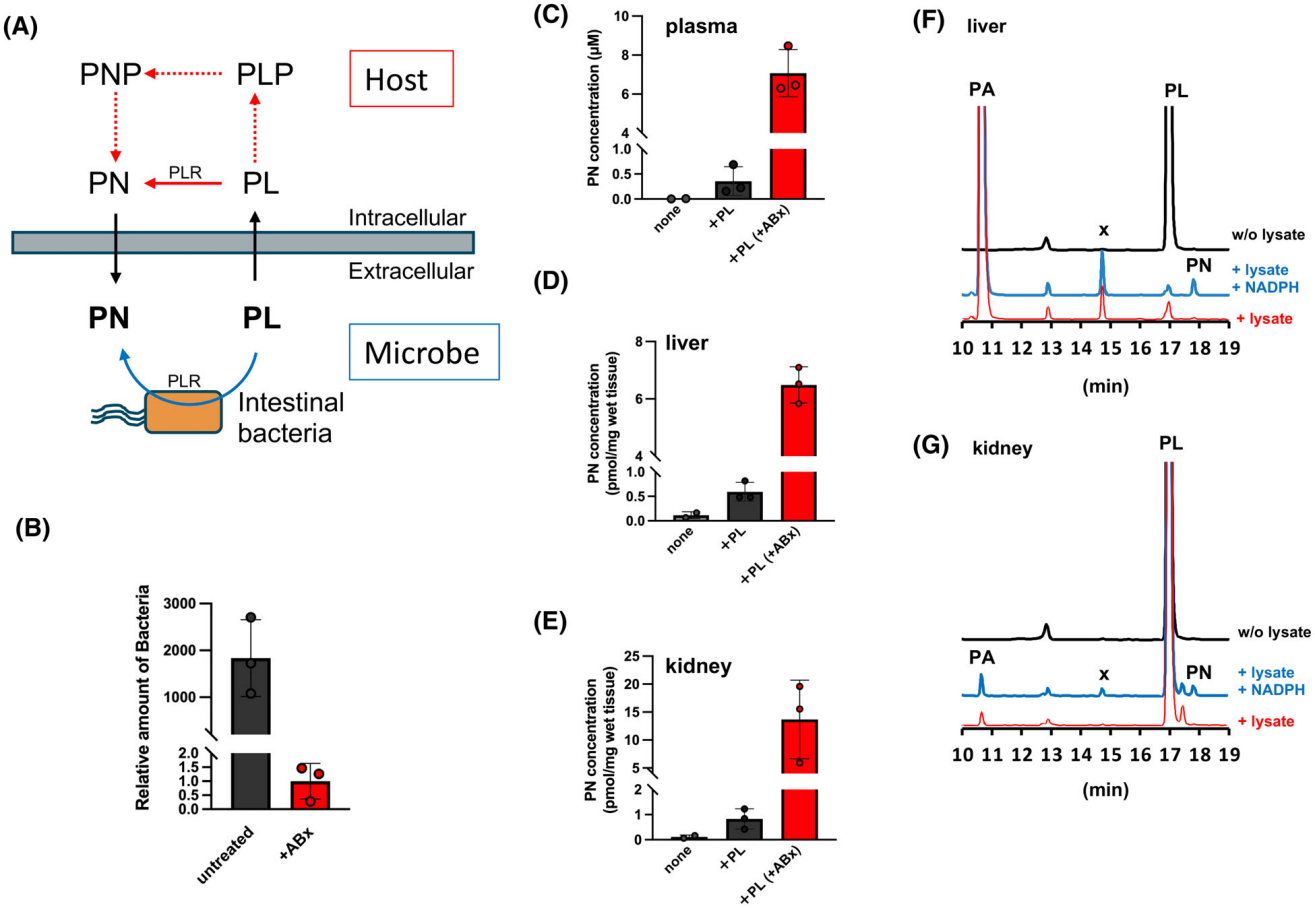
Host enzyme(s) mediate the conversion of pyridoxal (PL) to pyridoxine (PN) in mice. (A) Schematic illustration of possible pathways for PN production from PL. PN may be generated through endogenous host pathways via a direct reduction of PL or a sequential PL → PLP → PNP → PN pathway (red lines). Alternatively, PN production could result from gut microbiota‐mediated conversion of PL (blue line). (B) Effect of antibiotic (ABx) treatment on gut microbiota abundance. Mice were treated with a broad‐spectrum antibiotic cocktail to deplete intestinal microbiota. The relative abundance of gut microbiota was estimated by quantifying 16S rRNA gene copy number in fecal samples from ABx‐treated mice (+ABx) and untreated control mice (none), confirming effective microbiota depletion. Data are presented as mean ± SD (*n* = 3 mice). (C–E) Impact of gut microbiota depletion on PL → PN conversion. Untreated or ABx‐treated mice received oral PL (0.4 mL of 10 mg·mL^−1^) [+PL or +PL (+ABx)]. Untreated mice receiving water served as controls (none). Plasma, liver, and kidney samples were collected 1 h after administration, and PN concentrations were measured by high‐performance liquid chromatography (HPLC). PN levels in plasma (C), liver (D), and kidney (E) were not decreased in the ABx‐treated mice, indicating that PL‐to‐PN conversion occurs independently of the gut microbiota. Data are presented as mean ± SD (*n* = 3 for PL‐administrated mice and *n* = 2 for water‐administrated mice). (F, G) PL reduction activity in tissue extracts. Liver (F) and kidney (G) extracts were incubated with PL in the presence or absence of NADPH, followed by vitamin B_6_ vitamer analysis by HPLC. Shown are representative chromatograms from *n* = 3 independent experiments, all of which exhibited comparable elution profiles, demonstrating NADPH‐dependent conversion of PL to PN. An unknown metabolite (compound X; retention time 14.7 min) was detected and subsequently identified as 4‐pyridoxolactone (4‐PLA).

We previously identified the first bacterial pyridoxal reductase (PLR), PdxI, in *E. coli*, which exhibits strong PLR activity, enabling *E. coli* to effectively convert extracellular PL into PN [35]. Homologs of the *E. coli* PLR are present in various intestinal bacteria, suggesting that gut microbiota might contribute to the PL‐to‐PN conversion (Fig. 3A). To assess this, we repeated the experiments in antibiotic‐treated mice (ABx‐mice), which exhibit an approximately 2000‐fold reduction in gut microbiota compared with controls (Fig. 3B). Importantly, PN formation was still observed in ABx‐mice, with even higher levels detected in the plasma, liver, and kidney compared with the untreated mice (Fig. 3C–E). These results indicate that the PL‐to‐PN conversion in mice is predominantly mediated by host enzyme(s) rather than gut microbes. The elevated PN levels may reflect increasing intestinal PL absorption, as intracellular PL concentrations were also higher in the ABx‐treated mice (Fig. S1).

Two potential intracellular routes could explain PN formation from PL: (i) direct reduction of PL to PN by PLR (Fig. 3A, red solid line) or (ii) an indirect pathway involving phosphorylation, reduction, and dephosphorylation (PL → PLP → PNP → PN) (Fig. 3A, red broken lines). However, neither of the key reactions, PL reduction nor PLP reduction, has been previously detected in mammals. To elucidate the step(s) involved in PN formation, we measured enzyme activities in cell lysates prepared from mouse liver and kidney. Cell‐free extracts were incubated with PL or PLP in the presence or absence of NADPH, and the resulting mixtures were analyzed by high‐performance liquid chromatography (HPLC). In liver extracts, most PL was converted to PA regardless of NADPH, likely via aldehyde oxidase (AOX). Importantly, a small but significant amount of PN was produced only when NADPH was present (Fig. 3F). A similar NADPH‐dependent PN formation was observed in kidney extracts (Fig. 3G). When PLP served as the substrate, PN was not detected, indicating that the reduction step specifically targets PL rather than PLP (Fig. S2). Collectively, these findings provide evidence that NADPH‐dependent PLR(s) are probably responsible for the PL‐to‐PN conversion in mammals.

### Human AKR1C1 catalyzes NADPH‐dependent PL → PN conversion

All previously characterized PLRs from fungi, plants, and bacteria belong to the AKR superfamily [33, 34, 35, 36]. AKR superfamily enzymes are widely distributed across all domains of life and catalyze the reduction of carbonyl groups in diverse substrates [38, 39]. Humans possess 15 AKR isozymes (AKR1A1, AKR1B1, AKR1B10, AKR1B12, AKR1C1, AKR1C2, AKR1C3, AKR1C4, AKR1D1, AKR1E2, AKR6A3, AKR6A5, AKR6A9, AKR7A2, and AKR7A3) and play roles in various biological processes [37]. Although these proteins share only weak sequence identity with known PLRs (~20%) and lack clear phylogenetic relationships (Fig. S3A), several AKRs exhibit activity toward compounds structurally related to PL. For example, members of the AKR1A, AKR1B, and AKR7A subfamilies reduce pyridine aldehyde isomers, chlorobenzaldehydes, and carboxybenzaldehydes (Fig. S3B) [40], raising the possibility that certain AKRs may catalyze PL → PN conversion.

**Fig. 4:**
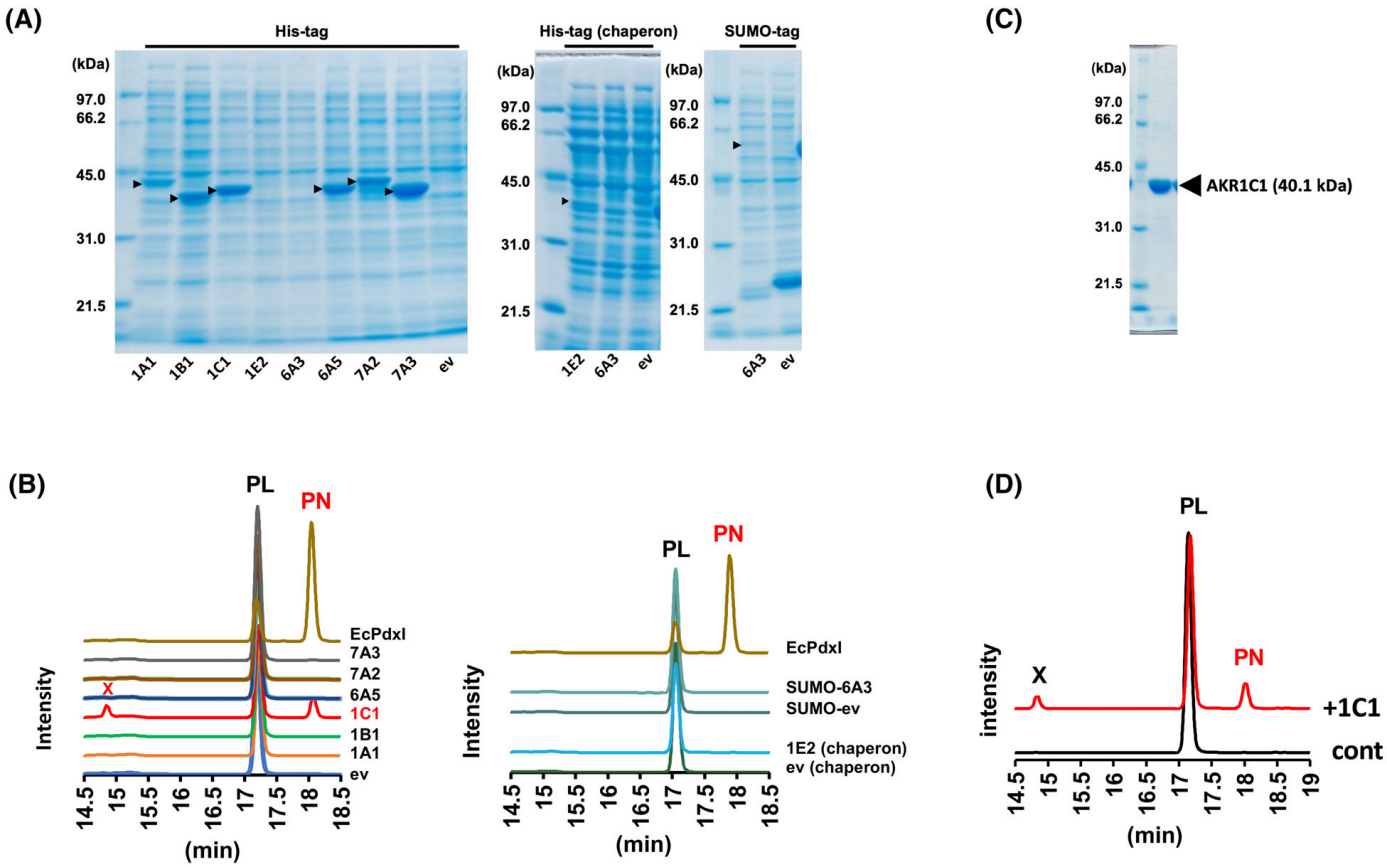
Expression and purification of human Aldo‐Keto Reductase (AKR) isozymes and their reactivity toward pyridoxal (PL). (A) Expression of human AKR isozymes in *E. coli*. Human AKR isozymes were heterologously expressed in *E. coli* BL21 Star™ (DE3) as N‐terminal His‐tagged proteins. AKR1A1, AKR1B1, AKR1C1, AKR6A5, AKR7A2, and AKR7A3 were obtained as soluble proteins (indicated by triangles). AKR1E2 and AKR6A3 required co‐expression with molecular chaperones or expression as *N*‐terminal SUMO‐tagged proteins to improve solubility. (B) Reactivity of *E. coli* cell‐free extracts expressing each AKR isozyme with PL. Cell‐free extracts prepared from *E. coli* expressing individual AKR isozymes were incubated with PL in the presence of NADPH, and reaction products were analyzed by high‐performance liquid chromatography (HPLC). Extracts expressing AKR1C1 showed PL‐to‐PN conversion and produced an additional metabolite (compound X; RT 14.7 min). An *E. coli* lysate expressing PdxI was included as a positive control. (C) SDS/PAGE analysis of purified AKR1C1. AKR1C1 was purified by affinity chromatography, and protein purity was assessed by SDS/PAGE, showing a predominant band corresponding to the expected molecular mass of AKR1C1. (D) Reactivity of purified AKR1C1 with PL. Purified AKR1C1 was incubated with PL in the presence of NADPH, and reaction products were analyzed by HPLC. The enzyme catalyzed the formation of pyridoxine (PN) and compound X, confirming that AKR1C1 directly mediates PL conversion. Compound X was later identified as 4‐PLA. (A–D) Shown is a representative result from at least three independent experiments, all of which yielded comparable profiles.

To test this hypothesis, we selected eight human AKRs (AKR1A1, AKR1B1, AKR1C1, AKR1E2, AKR6A3, AKR6A5, AKR7A2, and AKR7A3) for functional screening. Each protein was expressed in *E. coli* as N‐terminal His‐tag or small ubiquitin‐related modifier (SUMO)‐tag fusion proteins. After optimization of expression conditions, all target proteins were expressed as soluble forms (Fig. 4A). The *E. coli* cell lysates containing the individual AKRs were incubated with PL and NADPH, and the reaction mixtures were analyzed by HPLC. Strikingly, only AKR1C1‐expressing lysate exhibits PN‐forming activity (Fig. 4B). In addition, an unidentified compound (designated as compound X; retention time 14.7 min) was detected in the same reaction (Fig. 4B). Substitution of PL by PLP did not generate PN, PNP, or compound X. Assay with purified AKRs confirmed that AKR1C1 alone catalyzes PN formation from PL, along with production of compound X (Fig. 4C,D).

**Fig. 5:**
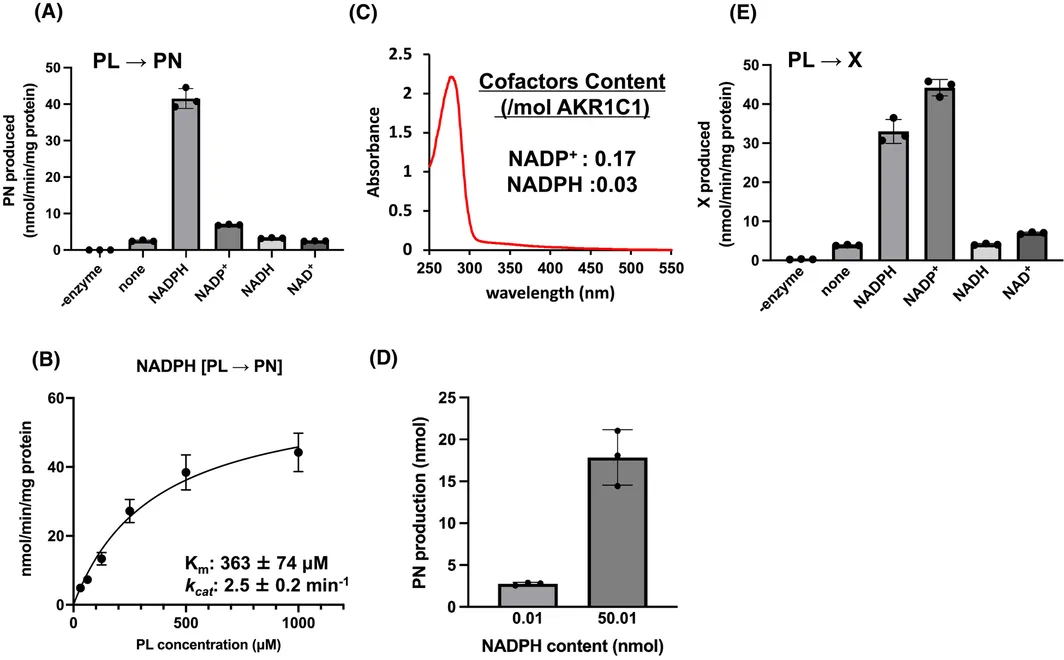
Cofactor dependency and kinetic parameters of AKR1C1‐mediated reactions. (A) Cofactor dependency of PL → PN conversion. Reactions were performed with 500 μm pyridoxal (PL) and 200 μm of various cofactors to assess cofactor specificity for PL reduction. (B) Kinetic analysis of pyridoxal reductase (PLR) activity. PLR activity was measured in the presence of 0.2 mm NADPH. For each substrate concentration, initial reaction rates were measured in triplicate and data are presented as mean ± SD. Kinetic parameters were obtained by fitting the data to the Michaelis–Menten equation using GraphPad Prism 10. (C) UV–visible absorption spectrum and bound cofactor content of purified AKR1C1. The amounts of enzyme‐bound cofactors were quantified using an NADP/NADPH Assay Kit‐WST (Dojindo, Kumamoto, Japan), revealing 0.17 mol of NADP^+^ and 0.03 mol of NADPH per mol of AKR1C1. (D) Pyridoxine (PN) formation by AKR1C1 in the absence of exogenous NADPH. A reaction mixture containing 50 nmol PL and 0.46 nmol purified AKR1C1 was incubated with or without addition of 50 nmol NADPH. In the absence of added NADPH—where only 0.01 nmol of tightly bound NADPH was present—2.8 nmol of PN was produced. (E) Cofactor dependency of PL‐to‐compound X conversion. Reactions were performed with 500 μm PL and 200 μm of indicated cofactors to assess the cofactor specificity of PL‐to‐compound X formation. This activity, later identified as PL dehydrogenase (PLD) activity, showed a distinct cofactor requirement from PLR activity. Data in panels other than (C) are presented as mean ± SD from three independent experiments.

AKR1C1 showed the highest PN‐forming activity in the presence of NADPH (Fig. 5A). Neither the reverse reaction (NADP^+^‐dependent PN oxidation to PL) nor NADPH‐dependent PLP reduction was detected under any condition (data not shown). Note that the presence of an N‐terminal His‐tag did not affect PN or compound X production (Fig. S4). Collectively, these findings identify AKR1C1 as the first mammalian PLR, catalyzing NADPH‐dependent reduction of PL to PN.

### Unique characteristics of AKR1C1‐mediated PL metabolism

AKR1C1, also called 3α‐hydroxysteroid dehydrogenase, is well‐characterized for its role in steroid hormone metabolism. The enzyme catalyzes NADPH‐dependent reductions at various positions on the steroid nucleus, acting as a 3‐, 17‐, and 20‐ketosteroid reductase [41, 42]. Among these substrates, AKR1C1 exhibits the highest activity toward progesterone, converting it to its inactive form, 20‐alpha‐hydroxy‐progesterone [41, 42]. AKR1C1 also reduces reactive aldehydes, such as 4‐hydroxy‐2‐nonenal and other α, β‐unsaturated aldehydes produced during lipid peroxidation, contributing to cellular defense against oxidative stress [43, 44].

Kinetic parameters for AKR1C1‐catalyzed PL reduction to PN were determined under saturating NADPH conditions. The reaction followed Michaelis–Menten kinetics over the tested PL concentration range (0–1 mm), yielding the following parameters: *K*_m_ = 363 ± 74 μm, *k*_cat_ = 2.5 ± 0.2 min^−1^, and *k*_cat_/*K*_m_ = 6.9 min^−1^·mm ^−1^ (Fig. 5B, Table 1). Although this catalytic efficiency is several orders of magnitude lower than that of the bacterial pyridoxal reductase *E. coli* PLR (PdxI: *k*_cat_/*K*_m_ ~ 10^5^ min^−1^·mm ^−1^) [35, 36], it is within one order of magnitude of that reported for *S. pombe* PLR (*k*_cat_/*K*_m_ ~ 62 min^−1^·mm ^−1^) [33]. Among the known endogenous substrates of AKR1C1, the catalytic efficiency for PL reduction was lower than that for progesterone (*k*_cat_/*K*_m_ = 109 min^−1^·mm ^−1^), but comparable to those for androsterone (*k*_cat_/*K*_m_ = 9 min^−1^·mm ^−1^) and testosterone (*k*_cat_/*K*_m_ = 1.1 min^−1^·mm ^−1^) [42], suggesting that PL reduction may occur under physiological conditions.

**Table 1:** Kinetic parameters of AKR1C1‐catalyzed PLR (PL → PN) and PLD (PL → 4‐PLA) activities. The AKR1C‐catalyzed PLR and PLD activities were determined as described in the Materials and Methods section. The reaction mixture contained 100 mm sodium phosphate buffer (pH 7.0), 0.2 mm NADPH (for PLR activity) or NADP^+^ (for PLD activity), 10–1000 μm PL, and ~20 μg of AKR1C1 enzyme. For each substrate concentration, initial reaction rates were measured in triplicate. Kinetic parameters were obtained by fitting the data to the Michaelis–Menten equation using GraphPad Prism 10.

Enzyme	Activity	*K*_m_ (μm)	*k*_cat_ (min^−1^)	*k*_cat_/*K*_m_ (min^−1^·mm ^−1^)
AKR1C1	PLR	363 ± 74	2.5 ± 0.2	6.9
	PLD	629 ± 122	3.4 ± 0.3	5.4
AKR1C3	PLR	298 ± 53	0.32 ± 0.03	1.1
	PLD	1020 ± 448	0.76 ± 0.24	0.8
AKR1C4	PLR	–	Trace	–
	PLD	304 ± 65	0.36 ± 0.04	1.2

Enzymatic analysis revealed several unique properties of AKR1C1. First, the enzyme exhibited weak but significant PN‐forming activity even in the absence of exogenous NADPH (Fig. 5A). While PL to PN conversion was initially assumed to involve hydride transfer from NADPH, the stoichiometry between PN production and NADPH consumption was inconsistent. For instance, incubation of 0.46 nmol of AKR1C1, which contained 0.01 nmol NADPH (Fig. 5C), with PL produced 2.8 nmol PN—approximately 280‐fold more than the available NADPH content (Fig. 5D). This suggests an apparent cofactor‐independent mechanism or recycling of reducing equivalents.

Second, AKR1C1 catalyzed the formation of an additional compound, compound X, from PL (Fig. 4D). The compound X accumulated without detectable degradation and was generated exclusively from PL, with no production observed from PLP or PN. These observations indicated that compound X is neither an intermediate of the PL → PN pathway nor a PN metabolite.

Third, compound X formation displayed a unique cofactor dependency. NADP^+^ markedly enhanced compound X production, and NADPH also supported formation at a comparable level (Fig. 5E). Interestingly, low but detectable levels of compound X were produced even without added cofactors, suggesting a mechanism that may differ from conventional AKR reactions.

Taken together, these findings highlight unique features of AKR1C1: its apparent ability to catalyze PL‐to‐PN conversion without exogenous NADPH and its capacity to generate compound X from PL via a mechanism that may differ from canonical NADP^+^/NADPH‐dependent reactions.

### Identification of compound X as 4‐pyridoxolactone

**Fig. 6:**
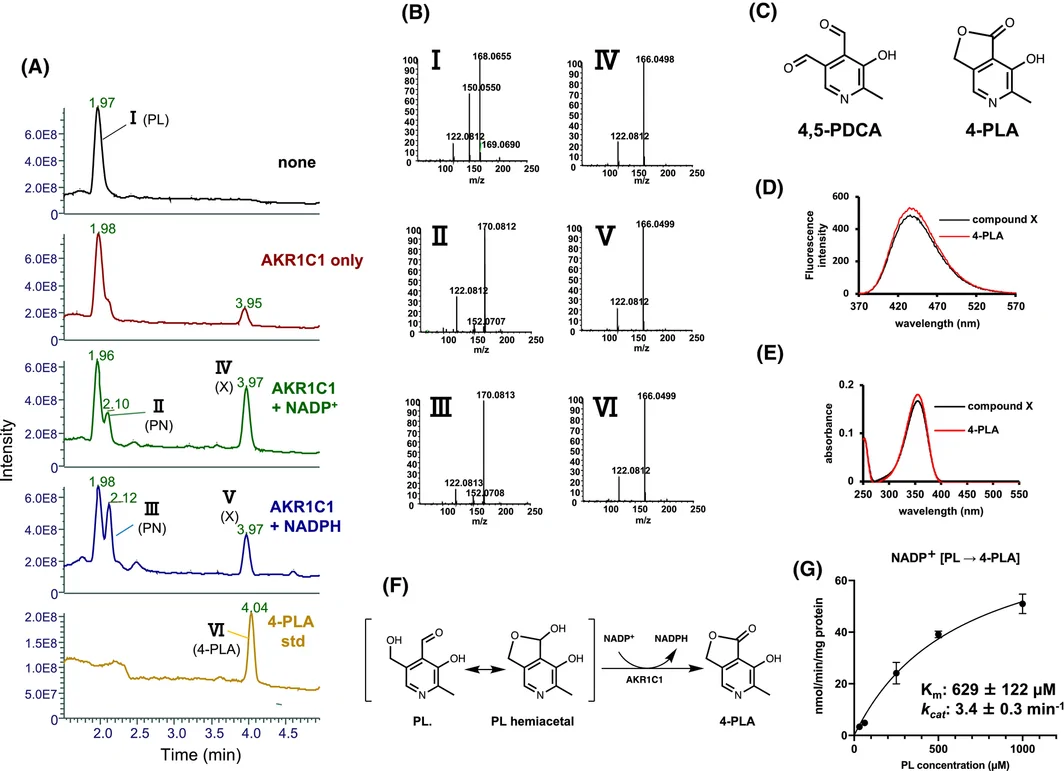
AKR1C1 catalyzes NADP^+^‐dependent conversion of pyridoxal (PL) to 4‐pyridoxolactone (4‐PLA). (A) LC–MS analysis of AKR1C1‐mediated reaction with PL. Purified AKR1C1 was incubated with 250 μm PL in the presence or absence of NADPH or NADP^+^, and reaction products were analyzed by LC–MS. Two new peaks (RT: 2.12 min and 3.97 min) appeared in reactions containing either NADPH or NADP^+^. An authentic 4‐PLA standard was analyzed under identical conditions for comparison. (B) MS spectra of detected peaks. MS spectra corresponding to Peaks I–VI are shown. Peak I corresponded to PL, and Peaks II and III were derived from pyridoxine (PN). Peaks IV and V, attributed to compound X, showed MS spectra identical to those of the 4‐PLA standard. (C) Proposed structures of compound X based on MS analysis. Two candidate structures—3‐hydroxy‐2‐methylpyridine‐4,5‐dicarbaldehyde (4,5‐PDCA) and 4‐PLA—were considered; subsequent analyses supported identification as 4‐PLA. (D, E) Spectroscopic characterization of compound X. UV–vis (D) and fluorescence spectra (E; excitation at 360 nm) of the compound X isolated by high‐performance liquid chromatography (HPLC) (black lines) are shown. Both spectra matched those of the authentic 4‐PLA standard (red lines), confirming the identity of compound X as 4‐PLA. (F) AKR1C1‐catalyzed PL dehydrogenase (PLD) activity (PL → 4‐PLA). Schematic representation of AKR1C1‐mediated oxidation of PL to 4‐PLA using NADP^+^ as a cofactor. (G) Kinetic parameters of PLD activity. PLD activity was assayed in the presence of 0.2 mm NADP^+^. For each substrate concentration, initial reaction rates were measured in triplicate and data are presented as mean ± SD. Kinetic parameters were calculated by fitting data to the Michaelis–Menten equation using GraphPad Prism 10. (A, B, D, E) Shown are representative results from at least two independent experiments, all of which yielded comparable profiles.

To identify compound X, the reaction products of AKR1C1 with PL were analyzed using LC–MS. Under our experimental conditions, PL eluted at RT 1.98 min (m/z 168.0655, theoretical [M + H]^+^ 168.065), while two products appeared at RT 2.12 min and 3.97‐min (Fig. 6A). The 2.12‐min peak corresponded to PN (observed m/z 170.0812, theoretical [M + H]^+^ 170.081) (Fig. 6B, III), whereas the 3.97‐min peak was assigned to compound X. The mass spectrum of compound X displayed an [M + H]^+^ ion at m/z 166.499 (Fig. 6B, V), indicating a loss of two hydrogen atoms from PL.

Two candidate structures for compound X, 3‐hydroxy‐2‐methylpyridine‐4,5‐dicarbaldehyde (4,5‐PDCA) or 4‐pyridoxolactone (4‐PLA), were considered (Fig. 6C). If compound X were 4,5‐PDCA, its reduction should yield PN. However, no PN was detected after sodium borohydride (NaBH_4_) treatment, demonstrating the absence of aldehyde group(s) (Fig. S5). 4‐PLA is known to exhibit an absorption at ~355 nm and a strong fluorescence upon excitation at 360 nm. The UV–Vis and fluorescence spectra of compound X, isolated by HPLC, were identical to those of the authentic 4‐PLA (Fig. 6D,E). In addition, compound X and the 4‐PLA standard displayed identical LC–MS profiles (Fig. 6A,B, see peaks of IV, V and VI). All data supported the conclusion that compound X is 4‐PLA.

To date, PL dehydrogenase (PLD), which catalyzes PL → 4‐PLA conversion, has been identified in *Microbacterium luteolum* and *Mesorhizobium loti*. The PLD from *M. loti* belongs to the short‐chain dehydrogenase/reductase (SDR) family, whereas PLD from *M. luteolum* is an AKR superfamily member (AKR15A) [45, 46]. Despite their structural differences, both enzymes catalyze NAD^+^‐dependent hydride transfer from PL hemiacetal, which is generated via nonenzymatic intramolecular reaction, to form 4‐PLA [47]. By analogy, we speculated that AKR1C1 catalyzes NADP^+^‐dependent PL dehydrogenation via hydride transfer from PL hemiacetal (Fig. 6F). Indeed, in the presence of NADP^+^, AKR1C1‐catalyzed PL → 4‐PLA conversion followed Michaelis–Menten kinetics in the 0–1 mm PL concentration range, with kinetic parameters of *K*_m_ = 629 ± 122 μm, *k*_cat_ = 3.4 ± 0.3 min^−1^, and *k*_cat_/*K*_m_ = 5.4 min^−1^·mm ^−1^ (Fig. 6G, Table 1). Although NADPH also supported 4‐PLA production, the reaction did not exhibit Michaelis–Menten behavior (Fig. S6), suggesting that NADPH is not the primary cofactor for this reaction. Note that the reverse reaction, that is, NADPH‐dependent 4‐PLA → PL conversion, was not observed. These findings establish AKR1C1 as a bifunctional enzyme in PL metabolism, catalyzing both the NADPH‐dependent PL reduction to PN and NADP^+^‐dependent PL oxidation to 4‐PLA.

### Proposed mechanism of AKR1C1‐catalyzed PL → PN and PL → 4‐PLA conversion

**Fig. 7:**
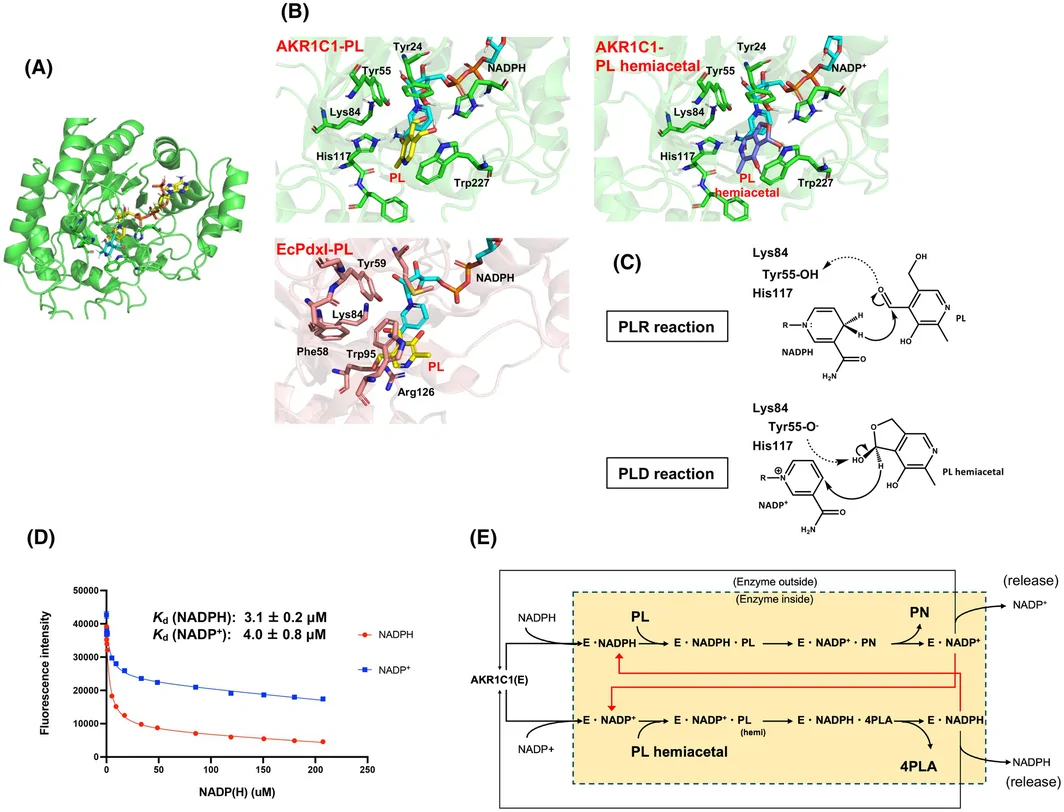
Predicted reaction mechanism of AKR1C1 for pyridoxal reductase (PLR) and PL dehydrogenase (PLD) activities. (A, B) Predicted binding modes of AKR1C1 with pyridoxal (PL) and 4‐PLA based on docking simulations. (A) Overall structure of the predicted AKR1C1–NADPH–PL complex. (B) Active site structure of the AKR1C1–NADPH–PL complex (left) and the AKR1C1–NADP^+^–PL hemiacetal complex (right). Structural representations were generated using PyMOL. (C) Proposed mechanisms of AKR1C1‐catalyzed PLR and PLD activities. In the reduction (PLR) reaction, a hydride ion is transferred from NADPH to the aldehyde group of PL, with Tyr55 acting as a proton donor (top). In the oxidation (PLD) reaction, Tyr55 deprotonates the hydroxyl group of the PL hemiacetal, and a hydride ion is transferred from the C4′ of the substrate to NADP^+^ (bottom). (D) Cofactor binding analysis of NADPH and NADP^+^ to AKR1C1. Cofactor binding was assessed by monitoring quenching of the intrinsic tryptophan fluorescence of AKR1C1 (0.5 μm) upon titration of NADPH or NADP^+^ (0–0.2 mm) in 50 mm sodium phosphate buffer (pH 7.0). (E) Proposed catalytic cycle of AKR1C1. AKR1C1 exhibits dual enzymatic activity toward PL: NADPH‐dependent PLR activity and NADP^+^‐dependent PLD activity. Due to its high affinity for NADP(H), the cofactor remains bound after a single reaction step, enabling continuous intramolecular recycling of NADP^+^ and NADPH during successive catalytic cycles (red arrows).

Docking simulations of AKR1C1 with PL and PL hemiacetal provided insight into the catalytic mechanisms. These models revealed a binding orientation favorable for hydride transfer, with the C4′ carbon and oxygen atom of the PL or 4‐PLA in close proximity to the C4 of NADP(H) and the hydroxyl group of the catalytic residue Tyr55, respectively (Fig. 7A,B). This predicted binding mode is markedly different from that of *E. coli* PdxI, whose crystal structure in complex with PL (PDB ID: 8TF1) shows a highly constrained substrate pocket in which PL is tightly sandwiched between Trp95 and Arg126. In PdxI, π‐stacking from Trp95 and hydrogen‐bonding interactions involving Arg126 rigidly constrain the orientation of PL within the active site [36]. Such interactions are absent in AKR1C1, which may explain the broader substrate specificity of AKR1C1 compared with PdxI (Fig. 7B). Based on this predicted binding mode, AKR1C1 likely catalyzes hydride transfer via a Tyr55‐mediated mechanism, consistent with catalytic mechanisms established for other AKR1C1 substrates (Fig. 7C) [36, 48].

AKR1C enzymes follow an ordered bi‐bi mechanism: NADPH binds first, followed by substrate binding, chemical transformation, product release, and finally NADP^+^ dissociation to complete the catalytic cycle (Fig. 7E, black lines). This cycle is governed by strong NADP(H) binding affinity and slow release of both cofactor and product [49]. Trp fluorescent titration experiments confirmed strong cofactor binding, with dissociation constants (*K*_d_) of 4.0 μm for NADP^+^ and 3.2 μm for NADPH (Fig. 7D). Furthermore, purified AKR1C1 retained ~0.2 mol NADP^+^ per mol enzyme after two chromatography steps (Ni^2+^ affinity and gel filtration), confirming tight cofactor binding (Fig. 5C).

This tight cofactor binding, together with the interconvertible nature of PL and PL hemiacetal, likely enables internal NADP(H) regeneration during catalytic cycle, allowing continuous enzymatic turnover without cofactor exchange (Fig. 7E, red lines). This mechanism explains several unusual features observed: apparent cofactor‐independent catalytic reaction, discrepancy between PN production and NADPH consumption, and dual cofactor dependence observed in PLD activity.

### Cross‐talk of AKR1C1 mediated metabolism and vitamin B_6_ availability

To investigate the physiological significance of AKR1C isozymes in vitamin B_6_ homeostasis, we overexpressed N‐terminal His‐tagged AKR1C1 in HEK293 cells and assessed its impact on B_6_ metabolism. HEK293 cells were selected as the host because they exhibit minimal endogenous AKR1C expression (https://www.proteinatlas.org/) and negligible basal activity for PL‐to‐PN conversion [31, 32]. Overexpression of AKR1C1 was confirmed by western blot analysis using an anti‐His‐tag antibody (Fig. S7).

**Fig. 8:**
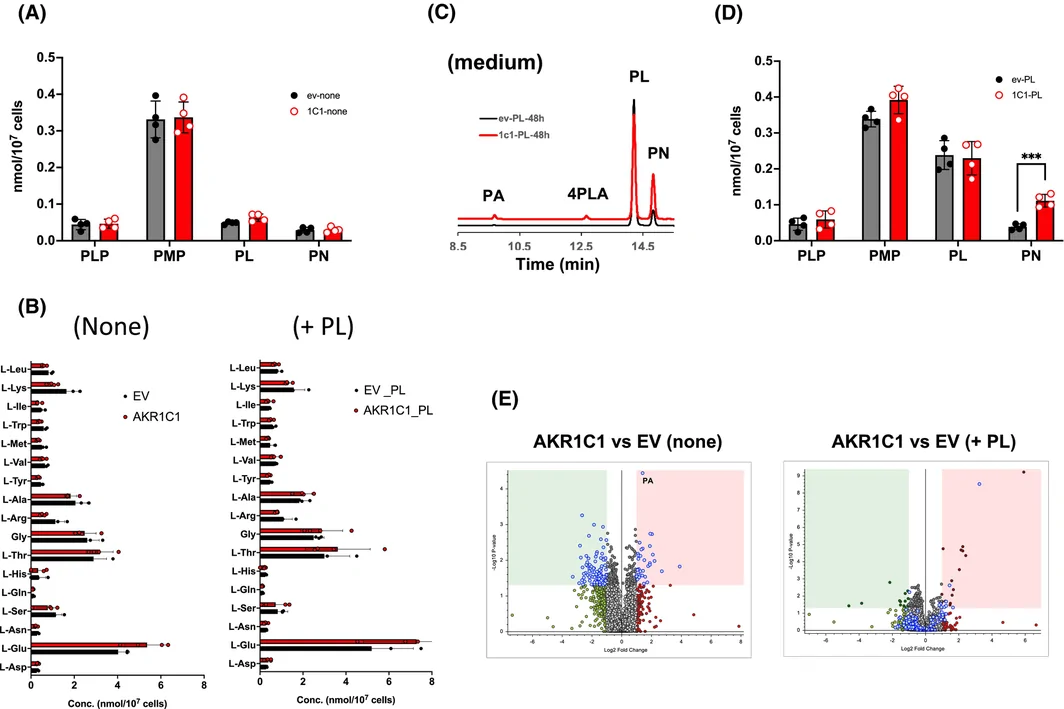
Effect of AKR1C1 overexpression in HEK293 cells on intracellular and extracellular vitamin B_6_ vitamer profiles. (A) Intracellular vitamin B_6_ vitamer profiles in HEK293 cells. HEK293 cells were transfected with either an empty vector or an AKR1C1‐overexpression vector and cultured in standard medium [Dulbecco's modified Eagle's medium (DMEM) supplemented with fetal bovine serum (FBS)]. Intracellular vitamin B_6_ vitamers were extracted and analyzed by high‐performance liquid chromatography (HPLC). (B) Intracellular amino acid profiles under pyridoxal (PL) supplementation. Intracellular amino acid profiles were determined for the same cell lines cultured in either standard medium (none) or PL‐supplemented medium (+PL). (C, D) Vitamin B_6_ vitamer profiles in extracellular and intracellular fractions. Vitamin B_6_ vitamers were analyzed in extracellular culture medium (C) and intracellular cell extracts (D). AKR1C1‐overexpressing cells exhibited elevated extracellular levels of pyridoxine (PN), 4‐PLA, and PA. Intracellularly, AKR1C1 overexpression led to a marked increase in PN levels. Asterisks indicate statistically significant differences compared with empty‐vector controls. (E) Global metabolomic analysis of AKR1C1‐overexpressing HEK293 cells. Metabolomic profiling was performed on HEK293 cells transfected with empty vector or AKR1C1‐overexpression vector and cultured in either standard medium (none) or PL‐supplemented medium (+PL). Metabolites showing significantly altered intracellular concentrations in AKR1C1‐overexpressing cells compared with empty vector controls under standard conditions are highlighted with blue circles. These AKR1C1‐driven metabolic changes were not observed under PL‐supplemented conditions. Data in panels other than (C) and (E) represent mean ± SD from four independent experiments.

Under standard culture conditions, AKR1C1 overexpression did not significantly affect intracellular B_6_ vitamer pools (Fig. 8A) nor disrupt amino acid homeostasis (Fig. 8B, left). In contrast, supplementation with 100 μm PL in the medium induced a pronounced shift of B_6_ vitamer profile: the AKR1C1‐overexpressing cells converted approximately 30% of extracellular PL to PN and accumulated detectable levels of 4‐PLA and PA (Fig. 8C). Intracellular PN levels also increased markedly (Fig. 8D). These data demonstrated that under high PL conditions, AKR1C1 overexpression enhances PLR and PLD activity and redirects B_6_ flux toward PN and 4‐PLA.

Metabolomic analysis uncovered previously unrecognized cross‐talk between AKR1C1‐mediated metabolism and vitamin B_6_ availability. Under standard conditions, AKR1C1 overexpression altered several metabolites, including a notable increase in PA. Many of the affected compounds remained unidentified (Fig. 8E, left, blue circles), likely reflecting the enzyme's broad substrate specificity. Notably, these AKR1C1‐driven metabolic changes were absent under PL‐supplemented conditions (Fig. 8E, right, blue circles), suggesting a potential competitive inhibition of AKR1C1‐mediated reactions by PL. Consistent with this observation, both PLR and PLD activities were inhibited by low concentrations of progesterone (Fig. S8).

Collectively, these results demonstrate that AKR1C1 contributes to vitamin B_6_ metabolism and highlight a mutual interaction between AKR1C1‐mediated aldehyde/keto acid metabolisms and vitamin B_6_ availability.

### AKR1C isozymes also act as bifunctional PLR and PLD

Humans possess four closely related AKR1C isozymes—AKR1C1, AKR1C2, AKR1C3, and AKR1C4—which share at least 84% primary sequence identity. These enzymes are expressed at varying levels across different organs and exhibit distinct reactivities toward keto/hydroxy steroids [42]. We purified recombinant AKR1C3 and AKR1C4 proteins and evaluated their PLR and PLD activities. Among these isozymes, AKR1C1 displayed the highest PLR activity (*k*_cat_/*K*_m_: 6.9 min^−1^·mm ^−1^), followed by AKR1C3 (*k*_cat_/*K*_m_: 1.1 min^−1^·mm ^−1^), while AKR1C4 showed weak PLR activity. For PLD activity, AKR1C1 was again most active (*k*_cat_/*K*_m_: 5.4 min^−1^·mm ^−1^), followed by AKR1C4 (*k*_cat_/*K*_m_: 1.2 min^−1^·mm ^−1^) and AKR1C3 (*k*_cat_/*K*_m_: 0.8 min^−1^·mm ^−1^) (Table 1).

*In vivo* PLR and PLD activities of AKR1C isozymes were further confirmed with an *E. coli* expression system. Specifically, we employed an *E. coli* BL21(DE3) strain that does not encode *pdxI*, and intrinsic PLR and PLD activities were negligible under our assay conditions. Strains overexpressing AKR1C1 or AKR1C3 produced both PN and 4‐PLA in PL‐supplemented medium, whereas AKR1C4‐expressing strains generated only 4‐PLA (Fig. S9), consistent with *in vitro* catalytic profiles. These results demonstrate that AKR1C isozymes function as bifunctional PLR/PLD enzymes *in vivo*, with isozyme‐specific differences in relative activity.

## Discussion

The canonical mammalian vitamin B_6_ salvage pathway has been attributed to three enzymes—PLK, PNPO, and PLPP. However, unexplained vitamin B_6_ profiles suggested additional contributors. In this study, we identified AKR1C isozymes as the fourth component of this pathway. These enzymes catalyze two previously unrecognized reactions: NADPH‐dependent reduction of PL to PN and NADP^+^‐dependent oxidation of PL to 4‐PLA (Fig. 1, red lines). This discovery expands the current framework of vitamin B_6_ metabolism and positions AKR1Cs as metabolic nodes linking B_6_ homeostasis with aldehyde and keto acid metabolism, including steroid hormones.

It remains unclear whether AKR1C1 isozymes serve as the primary PLR in mammals. Docking simulations demonstrated that AKR1C isozymes bind PL in an orientation favorable for hydride transfer (Fig. 7B,C), whereas other AKRs exhibit suboptimal binding geometries, positioning the C4′ of PL away from the NADPH C4 and/or the catalytic bases (Fig. S10). Cell‐based assays showed minimal PL‐to‐PN conversion activity in SH‐SY5Y, HEK293, and Caco‐2 cells, but robust activity in HepG2 cells [31, 32] (our unpublished results). This activity pattern correlates with AKR1C expression: strong expression of AKR1C isozymes has been reported in HepG2 cells [50, 51], and among the 15 human AKRs, only AKR1C isozymes display an expression profile consistent with PLR activity (Human Protein Atlas). Collectively, these findings strongly suggest that AKR1C isozymes may function as the primary PLR in mammals.

The AKR1C‐mediated PLD activity is NADP^+^‐dependent and was initially considered unlikely to occur *in vivo* due to the low intracellular NADP^+^ concentration [52]. However, we detected 4‐PLA production both in cultured cells (Fig. 8C, Fig. S9) and in mice following PL administration. In the latter cases, 4‐PLA production was confirmed by co‐chromatography with an authentic standard (Fig. S11), indicating that the AKR1C‐mediated PLD activity operates at the organismal level. Notably, mice possess eight AKR1C isozymes—AKR1C6, AKR1C12, AKR1C13, AKR1C14, AKR1C18, AKR1C19, AKR1C20, and AKR1C21. We confirmed that at least one of these, AKR1C6, exhibits both PLR and PLD activity *in vitro* (PLR activity: *k*_cat_/*K*_m_: 10 min^−1^·mm ^−1^, PLD activity: *k*_cat_/*K*_m_: 1.6 min^−1^·mm ^−1^, Fig. S12).

We show that AKR1C1 modulates vitamin B_6_ metabolism under high PL conditions. The relatively high *K*_m_ of AKR1C isozymes for PL (~0.3 mm) may restrict AKR1C activity to conditions of elevated PL availability, where other AKR1C1‐mediated metabolisms are attenuated (Fig. 8E). PLP is a modulator of steroid hormone receptor‐mediated gene expression [53]. The cross‐talk between vitamin B_6_ metabolism and AKR1C‐mediated may add a new regulatory layer linking vitamin B_6_ status to hormonal signaling and drug resistance.

The role of AKR1Cs in basal B_6_ metabolism remains unclear. In *E. coli*, PLR (PdxI) redirects PL metabolism from a direct phosphorylation pathway (PL → PLP) to a detoured pathway involving sequential conversion through PN and PNP (PL → PN → PNP → PLP), which serves as the primary PLP biosynthesis pathway from PL [35, 36]. Additionally, the presence of PdxI contributes to the attenuation of PL toxicity. Further investigation, including targeted knockout of AKR1C genes, may help clarify their contribution to PL metabolism in mammals. AKR1C isozymes exhibit tissue‐specific expression and are frequently overexpressed in certain cancers, such as nonsmall cell lung and prostate cancers, where they are implicated in tumor proliferation and drug resistance, including resistance to anthracyclines [37, 41, 44]. Variability in AKR1C1 expression across tissues and disease states may locally reshape B_6_ metabolism, influencing PLP availability, neurotransmitter synthesis, and amino acid metabolism—challenging the notion of a uniform B_6_ network.

Collectively, these findings support a broader model in which AKR1C isozymes act as metabolic hubs linking vitamin B_6_ metabolism, steroid hormone signaling, and drug resistance. This study reveals the involvement of a promiscuous enzyme family with variable expression in the vitamin B_6_ salvage pathway, highlighting the complexity of B_6_ metabolism and its regulation. It also underscores the need to revise the current framework of mammalian vitamin B_6_ metabolism to incorporate AKR1C‐mediated cross‐talk in the regulation of B_6_‐related physiological functions.

## Materials and methods

### Materials

PL, PN, PLP, NADPH, NADP^+^, antibiotics were obtained from Fujifilm Co. (Tokyo, Japan). 4‐PLA was from Toronto Research Chemicals (Toronto, Canada). Oligonucleotides were from Hokkaido System Science Co. (Sapporo, Japan). Codon‐optimized cDNA fragments for AKR proteins were from Invitrogen Japan (Tokyo, Japan) or GenScript Japan (Tokyo, Japan). Q5^®^ High‐fidelity DNA polymerase was from NEB Japan (Tokyo, Japan). Restriction enzymes were from Takara (Shiga, Japan). Other chemicals were of reagent grade and obtained from Fujifilm, Sigma‐Aldrich Japan Co. (Tokyo, Japan), Kanto Chemical (Tokyo, Japan), or Tokyo Kasei (Tokyo, Japan).

### Animal experiments

All animal experiment procedures were approved by the Animal Care Committee of the Nagoya University Graduate School of Bioagricultural Sciences (approval number: A230038). Six seven‐week‐old male ICR mice were purchased from Japan SLC Co. (Shizuoka, Japan). The mice were maintained in individual cage at 23 °C ± 2 °C with a 12‐h light/dark cycle (lights on at 8:00 AM), and were provided free access to food (CE‐2; CLEA Japan) and water. After 1 week of preliminary acclimation, the mice were randomly divided into two groups. Each group received one of the following treatments by oral administration: 0.5 mL of 10 mg·mL^−1^ PL or 0.5 mL of water. At 1.5 h post‐administration, blood, liver, and kidney samples were collected. Blood samples were anticoagulated with heparin and centrifuged. The resulting plasma was mixed with five volumes (v/v) of 0.8 m HClO_4_ and incubated on ice for 15 min. Subsequently, 2.5 volumes (v/v) of 0.8 m K_2_CO_3_ were added, followed by another 15‐min incubation on ice and centrifugation. The supernatant was collected and used as the plasma sample. Tissue samples were snap‐frozen in liquid nitrogen and stored at −80 °C until further analysis.

For antibiotic treatment experiments, an additional cohort of nine 7‐week‐old male ICR mice was purchased and acclimated in the same manner. Mice were randomly divided into two groups: one group received drinking water supplemented with an antibiotic cocktail (ampicillin, 0.5 g·L^−1^; vancomycin, 0.25 g·L^−1^; metronidazole, 0.5 g·L^−1^; and neomycin sulfate, 0.5 g·L^−1^) for 1 week, while the control group received untreated water. Following antibiotic treatment, fecal samples were collected. After a short fasting period, all mice were orally administered 0.5 mL of 10 mg·mL^−1^ PL. Blood, liver, and kidney samples were collected 1 h post‐administration and processed as described above. Mice fecal microbial DNA was extracted using the NucleoSpin DNA Stool Kit (Macherey‐Nagel). The number of microbiota was assessed by real‐time PCR using the extracted fecal DNA. The copy number of the bacterial 16S rRNA gene per quantity of extracted DNA was quantified by using primers 27‐fw (AGAGTTTGATCCTGGCTCAG) and 519‐rv (ATTACCGCGGCKGCTG) with KOD SYBR qPCR Mix (TOYOBO).

### Plasmid construction

The strains and plasmids used in this study are listed in Table 2. The nucleotide sequences of codon‐optimized human AKR proteins used in this study (AKR1A1, AKR1B1, AKR1C1, AKR1C3, AKR1C4, AKR1E2, AKR6A3, AKR6A5, AKR7A2, and AKR7A3) are listed in the Supplementary Materials and have ~20‐bp extensions at both 5′ and 3′ termini that are homologous to the pET‐His6‐Sumo‐TEV‐LIC or pET28a‐TEV vector.

**Table 2:** Strains and plasmids used in this study.

Strains or plasmids	Genotype or description	Source or references
Strains		
AE104	*E*. *coli* Top10	Invitrogen
AE157	*E*. *coli* BL21 Star™ (DE3)	Invitrogen
AEK140	AE157/pET28a‐TEV‐AKR1A1	This study
AEK141	AE157/pET28a‐TEV‐AKR1B1	This study
AEK142	AE157/pET28a‐TEV‐AKR1C1	This study
AEK174	AE157/pET28a‐TEV‐AKR1C3	This study
AEK175	AE157/pET28a‐TEV‐AKR1C4	This study
AEK143	AE157/pET28a‐TEV‐AKR1E2	This study
AEK144	AE157/pET28a‐TEV‐AKR6A3	This study
AEK145	AE157/pET28a‐TEV‐AKR6A5	This study
AEK146	AE157/pET28a‐TEV‐AKR7A2	This study
AEK147	AE157/pET28a‐TEV‐AKR7A3	This study
AEK148	AE157/pET28a‐TEV (ev)	This study
AEK161	AE157/pET28a‐TEV‐AKR1E2, pG‐KJE8	This study
AEK162	AE157/pET28a‐TEV‐AKR6A3, pG‐KJE8	This study
AEK163	AE157/pET28a‐TEV (ev), pG‐KJE8	This study
AEK8	AE157/pET‐His6‐SUMO‐AKR6A3	This study
AEK35	AE157/pET‐His6‐SUMO (ev)	This study
AEK33	AE157/pCA24N‐PdxI	This study
AEH10	AE157/pET28a‐TEV‐AKR1C6	This study
Plasmids		
pET28a‐TEV‐AKR1A1	Expresses N‐terminal His‐tagged AKR1A1	This study
pET28a‐TEV‐AKR1B1	Expresses N‐terminal His‐tagged AKR1B1	This study
pET28a‐TEV‐AKR1C1	Expresses N‐terminal His‐tagged AKR1C1	This study
pET28a‐TEV‐AKR1C3	Expresses N‐terminal His‐tagged AKR1C3	This study
pET28a‐TEV‐AKR1C4	Expresses N‐terminal His‐tagged AKR1C4	This study
pET28a‐TEV‐AKR6A3	Expresses N‐terminal His‐tagged AKR6A3	This study
pET28a‐TEV‐AKR6A5	Expresses N‐terminal His‐tagged AKR6A5	This study
pET28a‐TEV‐AKR7A2	Expresses N‐terminal His‐tagged AKR7A2	This study
pET28a‐TEV‐AKR7A3	Expresses N‐terminal His‐tagged AKR7A3	This study
pET28a‐TEV‐AKR1C6	Expresses N‐terminal His‐tagged AKR1C6	This study
pET28a‐TEV	pET28a based, empty vector	Laboratory collection
pET‐His6‐SUMO‐AKR6A3	Expresses N‐terminal His‐tagged SUMO‐AKR6A3 fusion protein	This study
pET‐His6‐SUMO	pET‐His6‐Sumo‐TEV‐LIC cloning vector (1S), empty vector	Addgene #29659
pG‐KJE8	Chaperon expression	Takara Co.
pCA24N‐pdxI	Expresses N‐terminal His‐tagged PdxI	ASKA clone
pCDNA3.1‐FLAG‐c	Empty vector	Gift
pCDNA3.1‐FLAG‐c‐AKR1A1	Expresses N‐terminal His‐tagged AKR1A1 in mammalian cells	This study
pCDNA3.1‐FLAG‐c‐AKR1C1	Expresses N‐terminal His‐tagged AKR1C1 in mammalian cells	This study

Strains and plasmids used in this study are listed in Table 2. Vector fragments were prepared by PCR using primer pairs (SUMO‐cloning 1: CCCACCAATCTGTTCTCTGTGAGC and SUMO‐cloning 2: CGGATCCGAATTCGAGCGCC or TEV‐cloning 1: GGCACCCTGAAAATACAAATTCTCG and TEV‐cloning 2: TGAGATCCGGCTGCTAACAAAGC) with either the pET‐His6‐Sumo‐TEV‐LIC or pET28a‐TEV vector as templates. The pET‐His6‐Sumo‐TEV‐LIC cloning vector (1S) was a gift from Scott Gradia (Addgene plasmid no.: 29659; http://n2t.net/addgene:29659; RRID:Addgene_29 659). Each AKR gene and the corresponding vector fragment were assembled using a ligation‐independent cloning method [54]. The resulting plasmids were transformed into *E. coli* Top10 and selected on LB agar plates containing kanamycin (Km). The inserted gene sequences were verified by DNA sequencing.

For expression in mammalian cells, the AKR1A1 and AKR1C1 genes were cloned into the pCDNA3.1‐FLAG‐c vector, which was a gift from Terunao Takahara (Nagoya University). Both genes were amplified by PCR using primers pCDNA‐cloning1 (CTGGCTAGCGTTTAAACTTAAGCTTACCATGAGCAGCAGCCATCATCATCATC) and pCDNA‐cloning2 (ACTGTGCTGGATATCTGCAGGCTTTGTTAGCAGCCGGATCTCA), with pET28a‐TEV‐AKR1A1 or pET28a‐TEV‐AKR1C1 as templates. The pCDNA3.1‐FLAG‐c vector was linearized with *Hind*III and *EcoR*I. The vector and PCR‐amplified insert fragments were assembled using the same ligation‐independent method [54].

### Expression and purification of AKR1C isozymes

To obtain purified AKR1C enzymes, BL21 Star™ (DE3) cells (Invitrogen Japan) harboring pET28a‐TEV‐AKR1C1 (AEK142), pET28a‐TEV‐AKR1C2 (AEK174), pET28a‐TEV‐AKR1C3 (AEK175), or pET28a‐TEV‐AKR1C6 (AEH10) were precultured overnight at 37 °C in LB medium supplemented with 30 μg·mL^−1^ Km and 0.2% glucose. Then, 1 mL of the preculture was inoculated into 200 mL of the AI medium (1.25% K_2_HPO, 0.23% KH_2_PO_4_, 1.2% polypeptone, 2.4% yeast extract, 0.5% glycerol, 0.05% glucose, 0.2% lactose) containing 100 μg·mL^−1^ Km and incubated at 30 °C with shaking for 20 h. Cells were harvested by centrifugation and resuspended in 10 volumes (v/w) of binding buffer (20 mm sodium phosphate, 0.5 m NaCl, 5 mm imidazole, pH 7.4). The cell suspension was disrupted by sonication, and cell debris was removed by centrifugation at 20000 ***g*** for 30 min at 4 °C. The resulting supernatant was applied to a HisTrap FF column (1 mL) (Cytiva, Tokyo, Japan), and His‐tagged AKR1C was purified by gradient elution using an elution buffer consisting of 20 mm sodium phosphate, 0.5 m NaCl, and 500 mm imidazole (pH 7.4). The buffer of purified protein was subsequently exchanged to a buffer containing 20 mm sodium phosphate and 0.5 m NaCl (pH 7.4) using a PD‐10 desalting column (Cytiva). The purified protein was stored at 4 °C until used.

### Enzyme assay

The PLR activity of cell‐free extract of *E. coli* strains expressing each of human AKR proteins was evaluated as follows: *E. coli* BL21 Star™ (DE3) cells harboring a plasmid (AEK140‐147 or AEK8) were precultured overnight in LB medium supplemented with appropriate antibiotics and 0.2% glucose at 37 °C, then transferred to 5 mL of AI medium and incubated until reaching the stationary phase at 30 °C. The chaperone plasmid pG‐KJE8 (Takara) was co‐introduced into the *E. coli* cells to increase the solubility of AKR1E2 (AEK161). Cells were harvested by centrifugation, resuspended in 500 μL of buffer containing 20 mm sodium phosphate (pH 7.0) and 0.5 m NaCl, and disrupted by sonication. After centrifugation at 20 000 ***g*** for 15 min at 4 °C, the resulting supernatant was used as the cell‐free extract for SDS/PAGE and activity assays.

The PLR and PLD activities of purified AKR1C isozymes were measured by HPLC. Reactions were performed in a 200 μL mixture containing 100 mm sodium phosphate buffer (pH 7.0), 0.2 mm NADPH or NADP^+^, 0.5 mm PL, and 20 μg of purified AKR1C enzyme. For kinetic parameter determination, reactions were conducted with varying PL concentrations (10, 50, 100, 250, 500, and 1000 μm). After incubation at 37 °C for 20 min, a 45‐μL aliquot of the reaction mixture was withdrawn, mixed with 5 μL of 8 M HClO_4_, and incubated on ice for 15 min to terminate the reaction. The mixture was then neutralized by adding 25 μL of 0.8 m K_2_CO_3_ and centrifuged at 20 000 ***g*** for 15 min at 4 °C. The resulting supernatant was diluted up to 50‐fold and subjected to HPLC analysis. To determine the *K*_m_ and V_max_ values, the initial rates of PN or 4‐PLA production were fitted to the Michaelis–Menten equation using nonlinear least‐squares regression analysis in GraphPad Prism 10 (GraphPad Software Inc., CA, USA). The *k*_cat_ value was calculated by dividing *V*_max_ by the molar concentration of AKR1C isozymes.

Mouse organs (liver, kidney, and testis) were homogenized in 5 volumes of (vol/mg wet weight) a buffer (100 mm sodium phosphate, pH 7.0, containing 1× protease inhibitor) and disrupted by sonication. After centrifugation, the supernatant was collected and used as a cell‐free extract for enzyme assays. Reactions were performed in a total volume of 400 μL containing 100 mm sodium phosphate (pH 7.0), 1× protease inhibitor (Nacharai Tesque, Kyoto, Japan) in the presence or absence of NADPH (100 μm) or PL or PLP (500 μm), to which half the volume of the cell‐free extract was added. To suppress the activity of aldehyde oxidase (AOX) activity, dissolved oxygen in the reaction mixture was removed by nitrogen bubbling prior to the reaction. The samples were incubated at 37 °C and the reaction was terminated using HClO_4_ and K_2_CO_3_ as described above. Samples were centrifuged and the resulting supernatants were diluted 50‐fold and analyzed by HPLC.

### HPLC analysis of B_6_ vitamers and amino acids

The concentrations of B_6_ vitamers were analyzed by HPLC as described previously [55]. Briefly, a 15‐μL aliquot of each sample was injected onto a Cosmosil PBr column (4.6 mm I.D. × 150 mm length, 3 μm particle size; Nacalai Tesque) equipped with a guard column (Mightysil RP‐18 GPII, 4.6 × 5 mm, 3 μm; Kanto Chemical, Tokyo, Japan). The mobile phase consisted of solvent A (33 mm phosphoric acid and 8 mm 1‐octanesulfonic acid, pH adjusted to 2.2 with KOH) and solvent B (80% [v/v] acetonitrile). Chromatographic separation was performed at a flow rate of 1.0 mL·min^−1^ with the column oven maintained at 28 °C. A post‐column reagent containing 1 mg·mL^−1^ sodium bisulfite in 1 m potassium phosphate buffer (pH 7.5) was used at a flow rate of 0.3 mL·min^−1^ to enhance the fluorescent intensity of PLP. Detection of B_6_ vitamers was carried out using fluorescence detection with an excitation wavelength of 328 nm and an emission wavelength of 393 nm.

The intracellular amino acid concentrations were determined as described previously [26, 27].

### LC–MS analysis

A 200‐μL reaction mixture containing 250 μm PL and purified AKR1C1 was incubated at 37 °C for 1 h in the presence or absence of 250 μm NADPH or NADP^+^. After incubation, 90 μL of the reaction mixture was mixed with 10 μL of 8 M HClO_4_ and incubated on ice for 15 min to stop the reaction. The mixture was then neutralized with 50 μL of 0.8 M K₂CO₃ to obtain the AKR1C1 reaction sample. The sample was centrifuged at 20 000 ***g*** for 15 min at 4 °C, and the supernatant was collected. The supernatant was filtered through a 0.45 μm filter and subjected to MS analysis. The AKR1C1 products were separated with the Vanquish Horizon UHPLC System with a Scherzo SM‐C18 column (Imtakt Corporation, Kyoto, Japan) using gradient elution of mobile phase A (5 mm ammonium formate +0.05% formic acid in water) and mobile phase B (0.3% formic acid in acetonitrile) at a flow rate of 0.2 mL·min^−1^. The gradient conditions were as follows: 0–4 min: 0–5%, 4–4.1 min: 5‐90%B, 4.1–7 min: 90%B, and 7–7.1 min: 90–0%B. Detection was carried out using an Orbitrap Exploris 240 mass spectrometer (Thermo Fisher Scientific, Waltham, MA, USA) equipped with a heated electrospray ionization (H‐ESI) source operating in positive ion mode. Other analysis conditions were as follows: Ion Source Type: H‐ESI, Spray Voltage: Static, Positive Ion: 3500 V, Negative Ion: 2500 V, Gas Mode: Static, Sheath Gas (Arb): 35, Aux Gas (Arb): 7, Sweep Gas (Arb): 0, Ion Transfer Tube Temperature: 320 °C, Vaporizer Temperature: 275 °C, APPI Lamp: Not in use.

The metabolites in HEK293 cells were extracted with MeOH/ACN/water (2/2/1) and sonication, and separated with an Intrada Amino Acid column (3 μm, 100 × 2 mm, Imtakt Corporation). The column temperature was maintained at 37 °C. The mobile phases consisted of Solvent A: 0.3% formic acid in water and Solvent B: acetonitrile/100 mm ammonium formate (20:80, v/v). The flow rate was set to 0.6 mL·min^−1^, and the injection volume was 5 μL. The gradient program was as follows: 0.00–4.00 min: 20% B, 4.00–14.00 min: linear increase to 100% B, 14.00–16.00 min: 100% B, 16.10–20.00 min: return to 20% B. Other analysis conditions were as follows: Spray voltage: 3500 V, Sheath gas: 60 arbitrary units, Auxiliary gas: 15 arbitrary units, Sweep gas: 2 arbitrary units, Ion transfer tube temperature: 350 °C, Vaporizer temperature: 350 °C.

Full MS scans were acquired at a resolution of 60 000 over an m/z range of 67–1000. Data‐dependent MS/MS (ddMS^2) was performed using higher‐energy collisional dissociation (HCD) with collision energies of 10%, 30%, and 50%, and Orbitrap resolution set to 15 000. Raw data were processed using Compound Discoverer 3.3 (Thermo Fisher Scientific, Waltham, MA, USA) in nontargeted analysis mode. Peak detection was performed with a minimum peak intensity threshold of 10 000. Ratios were generated for comparative analysis between experimental conditions.

### Docking simulation

Docking simulations were conducted using swissdock (https://www.swissdock.ch/) [56], employing the Attracting Cavities 2.0 algorithm. The receptor structures used in the simulations were derived from the crystal structure of AKR1C1 (PDB ID: 6IJX), with the bound ligand removed. The PL or PL hemiacetal binding site was defined based on the ligand‐binding pocket identified in the crystal structure, and the docking search was confined to a cubic region measuring 20 Å per side centered on this site. Figures showing the most favorable binding conformations with the lowest binding energies were generated using the PyMOL (https://www.pymol.org).

### Construction of AKR1C1‐overexpressing HEK293 cells

The HEK293 cell line (RRID:CVCL_0045) was obtained from the Japanese Collection of Research Bioresources (JCRB) Cell Bank. The cells were authenticated by the JCRB Cell Bank through Short Tandem Repeat (STR) profiling. All experiments were performed using cells that were confirmed to be negative for mycoplasma contamination. The HEK293 cells were cultured in Dulbecco's modified Eagle's medium (DMEM: Gibco, Waltham, MA, USA) supplemented with 10% fetal bovine serum (FBS: Life Technologies, Carlsbad, CA, USA), 100 U·mL^−1^ penicillin, and 100 μg·mL^−1^ streptomycin (Gibco). Cells were seeded in 10‐cm dishes and transfected at approximately 80% confluency with 5 μg of plasmid DNA using FuGENE6 transfection reagent (Promega, Madison, WI, USA) according to the manufacturer's protocol. Twenty‐four hours post‐transfection, PL was added at a final concentration of 0.1 mm. Cells were collected 48 h after PL treatment. The recombinant DNA experiments were conducted with the approval of the Biosafety Committee for Recombinant Organisms and Pathogenic Microorganisms at the Tokyo Metropolitan Institute of Medical Science (approval no.: 24‐020).

The AKR1C overexpression was confirmed by western blotting. Cells were lysed with 1 × SDS buffer and incubated at 65 °C for 10 min. For SDS/PAGE, 20 μL of lysates were loaded and separated on a 12% polyacrylamide gel, followed by transfer of proteins onto a PVDF membrane. The membrane was blocked with blocking one (Nacalai Tesque) and incubated with primary antibodies (1:1000 dilution) overnight at 4 °C. After three washes with PBST (PBS containing 0.1% Tween‐20), membranes were incubated with HRP‐conjugated secondary antibodies (5000‐fold dilution) for 2 h at room temperature, followed by three PBST washes. The following primary antibodies were used: anti‐β‐actin (A1978; Sigma, Aldrich, Japan), anti‐His‐tag (D291‐3; MBL, Nagoya, Japan), and anti‐AKR1A1 (15 054‐1‐AP; Proteintech Group, Rosemont, IL, USA). Signal detection was performed using an enhanced chemiluminescence (ECL) substrate and imaged with a ChemiDoc XRS^+^ (Bio‐Rad Japan, Tokyo, Japan).

## Author contributions

TI conceptualized the project and acquired funding. NK, YK, KI, KT, HN, NO, MA, and TI conducted the experiments and collected the data. NK, KT, MA, HH, and TI did the data analysis. NK and TI wrote the manuscript. All authors reviewed and edited the manuscript and have given approval to its final version.

## Conflict of interest

The authors declare no conflicts of interest associated with this manuscript.

## Supporting information


**Fig. S1.** Effect of gut microbiota on PL → PN conversion in mice.
**Fig. S2.** PN production activity in lysate of mouse organs.
**Fig. S3.** Phylogenetic analysis of human AKR proteins and their reactivity toward PL analogs.
**Fig. S4.** Effect of N‐terminal His‐tag on the reactivity of AKR1C1 toward PL.
**Fig. S5.** Identification of Compound X.
**Fig. S6.** Effect of NADPH and NADP^+^ on AKR1C1‐catalyzed PLR and PLD activities.
**Fig. S7.** Confirmation of AKR1C1 overexpression in HEK293 cells.
**Fig. S8.** Effect of progesterone on AKR1C1‐ and AKR1C3‐catalyzed PLR and PLD activities.
**Fig. S9.** Effect of expression of AKR isozymes on PL metabolism in *E. coli*.
**Fig. S10.** Predicted binding modes of AKR isozymes with PL based on docking simulations.
**Fig. S11.** Confirmation of PL‐to‐4‐PLA conversion in mice.
**Fig. S12.** Kinetic parameters of AKR1C6‐catalyzed PLR and PLD activities.

## Data Availability

The data that support the findings of this study are available on request from the corresponding author.

## References

[1] Reddy SK , Reynolds MS & Price JM (1958) The determination of 4‐pyridoxic acid in human urine. J Biol Chem 233, 691–696. 13575438

[2] Percudani R & Peracchi A (2003) A genomic overview of pyridoxal‐phosphate‐dependent enzymes. EMBO Rep 4, 850–854. 12949584 10.1038/sj.embor.embor914 PMC1326353

[3] Eliot AC & Kirsch JF (2004) Pyridoxal phosphate enzymes: mechanistic, structural, and evolutionary considerations. Annu Rev Biochem 73, 383–415. 15189147 10.1146/annurev.biochem.73.011303.074021

[4] Phillips RS (2015) Chemistry and diversity of pyridoxal‐5′‐phosphate dependent enzymes. Biochimica et Biophysica Acta (BBA) – Proteins and Proteomics 1854, 1167–1174. 25615531 10.1016/j.bbapap.2014.12.028

[5] Fitzpatrick TB *et al*. (2007) Two independent routes of *de novo* vitamin B6 biosynthesis: not that different after all. Biochem J 407, 1–13. 17822383 10.1042/BJ20070765

[6] Mukherjee T , Hanes J , Tews I , Ealick SE & Begley TP (2011) Pyridoxal phosphate: biosynthesis and catabolism. Biochimica et Biophysica Acta (BBA) – Proteins and Proteomics 1814, 1585–1596. 21767669 10.1016/j.bbapap.2011.06.018

[7] Whyte MP , Mahuren JD , Fedde KN , Cole FS , McCabe ER & Coburn SP (1988) Perinatal hypophosphatasia: tissue levels of vitamin B6 are unremarkable despite markedly increased circulating concentrations of pyridoxal‐5′‐phosphate. Evidence for an ectoenzyme role for tissue‐nonspecific alkaline phosphatase. J Clin Invest 81, 1234–1239. 3350970 10.1172/JCI113440 PMC329654

[8] Waymire KG , Mahuren JD , Jaje JM , Guilarte TR , Coburn SP & MacGregor G (1995) Mice lacking tissue non–specific alkaline phosphatase die from seizures due to defective metabolism of vitamin B–6. Nat Genet 11, 45–51. 7550313 10.1038/ng0995-45

[9] Di Salvo ML , Contestabile R & Safo MK (2011) Vitamin B6 salvage enzymes: mechanism, structure and regulation. Biochimica et Biophysica Acta (BBA) – Proteins and Proteomics 1814, 1597–1608. 21182989 10.1016/j.bbapap.2010.12.006

[10] Hanna MC , Turner AJ & Kirkness EF (1997) Human pyridoxal kinase. J Biol Chem 272, 10756–10760. 9099727 10.1074/jbc.272.16.10756

[11] Lee H‐S , Moon BJ , Choi SY & Kwon O‐S (2000) Human pyridoxal kinase: overexpression and properties of the recombinant enzyme. Mol Cells 10, 452–459. 10987144

[12] Musayev FN , Di Salvo ML , Ko T , Schirch V & Safo MK (2003) Structure and properties of recombinant human pyridoxine 5′‐phosphate oxidase. Protein Sci 12, 1455–1463. 12824491 10.1110/ps.0356203 PMC2323923

[13] Barile A , Graziani C , Antonelli L , Parroni A , Fiorillo A , di Salvo ML , Ilari A , Giorgi A , Rosignoli S , Paiardini A *et al*. (2024) Identification of the pyridoxal 5′‐phosphate allosteric site in human pyridox(am)ine 5′‐phosphate oxidase. Protein Sci 33, e4900. 38284493 10.1002/pro.4900 PMC10804683

[14] Jang YM , Kim DW , Kang T‐C , Won MH , Baek N‐I , Moon BJ , Choi SY & Kwon O‐S (2003) Human pyridoxal phosphatase. J Biol Chem 278, 50040–50046. 14522954 10.1074/jbc.M309619200

[15] Wilson MP , Plecko B , Mills PB & Clayton PT (2019) Disorders affecting vitamin B_6_ metabolism. J Inherit Metab Dis 42, 629–646. 30671974 10.1002/jimd.12060

[16] Ghatge MS , Al Mughram M , Omar AM & Safo MK (2021) Inborn errors in the vitamin B6 salvage enzymes associated with neonatal epileptic encephalopathy and other pathologies. Biochimie 183, 18–29. 33421502 10.1016/j.biochi.2020.12.025 PMC11273822

[17] Hadtstein F & Vrolijk M (2021) Vitamin B‐6‐induced neuropathy: exploring the mechanisms of pyridoxine toxicity. Advances in Nutrition 12, 1911–1929. 33912895 10.1093/advances/nmab033 PMC8483950

[18] Arai M , Yuzawa H , Nohara I , Ohnishi T , Obata N , Iwayama Y , Haga S , Toyota T , Ujike H , Arai M *et al*. (2010) Enhanced carbonyl stress in a subpopulation of schizophrenia. Arch Gen Psychiatry 67, 589. 20530008 10.1001/archgenpsychiatry.2010.62

[19] Tomioka Y , Numata S , Kinoshita M , Umehara H , Watanabe SY , Nakataki M , Iwayama Y , Toyota T , Ikeda M , Yamamori H *et al*. (2018) Decreased serum pyridoxal levels in schizophrenia: meta‐analysis and Mendelian randomization analysis. J Psychiatry Neurosci 43, 194–200. 29688875 10.1503/jpn.170053 PMC5915240

[20] Carvalho AF , Solmi M , Sanches M , Machado MO , Stubbs B , Ajnakina O , Sherman C , Sun YR , Liu CS , Brunoni AR *et al*. (2020) Evidence‐based umbrella review of 162 peripheral biomarkers for major mental disorders. Transl Psychiatry 10, 152. 32424116 10.1038/s41398-020-0835-5 PMC7235270

[21] Bucci L (1973) Pyridoxine and schizophrenia. Br J Psychiatry 122, 240. 10.1192/bjp.122.2.240 4714839

[22] Ananth JV , Ban TA & Lehmann HE (1973) Potentiation of therapeutic effects of nicotinic acid by pyridoxine in chronic schizophrenics. Can Psychiatr Assoc J 18, 377–383. 4147710 10.1177/070674377301800505

[23] Itokawa M , Miyashita M , Arai M , Dan T , Takahashi K , Tokunaga T , Ishimoto K , Toriumi K , Ichikawa T , Horiuchi Y *et al*. (2018) Pyridoxamine: a novel treatment for schizophrenia with enhanced carbonyl stress. Psychiatry Clin Neurosci 72, 35–44. 29064136 10.1111/pcn.12613

[24] Liu Z , Farkas P , Wang K , Kohli M & Fitzpatrick TB (2022) B vitamin supply in plants and humans: the importance of vitamer homeostasis. Plant J 111, 662–682. 35673947 10.1111/tpj.15859 PMC9544542

[25] Fitzpatrick TB (2024) B vitamins: an update on their importance for plant homeostasis. Annu Rev Plant Biol 75, 67–93. 38424064 10.1146/annurev-arplant-060223-025336

[26] Ito T , Yamamoto K , Hori R , Yamauchi A , Downs DM , Hemmi H & Yoshimura T (2019) Conserved pyridoxal 5’‐phosphate‐binding protein YggS impacts amino acid metabolism through pyridoxine 5′‐phosphate in *Escherichia coli* . Appl Environ Microbiol 85, e00430‐19. 30902856 10.1128/AEM.00430-19 PMC6532037

[27] Ito T , Hori R , Hemmi H , Downs DM & Yoshimura T (2020) Inhibition of glycine cleavage system by pyridoxine 5′‐phosphate causes synthetic lethality in *glyA yggS* and *serA yggS* in *Escherichia coli* . Mol Microbiol 113, 270–284. 31677193 10.1111/mmi.14415 PMC7060791

[28] Ito T (2022) Role of the conserved pyridoxal 5′‐phosphate‐binding protein YggS/PLPBP in vitamin B6 and amino acid homeostasis. Biosci Biotechnol Biochem 86, 1183–1191. 35803498 10.1093/bbb/zbac113

[29] Footitt EJ , Clayton PT , Mills K , Heales SJ , Neergheen V , Oppenheim M & Mills PB (2013) Measurement of plasma B_6_ vitamer profiles in children with inborn errors of vitamin B_6_ metabolism using an LC‐MS/MS method. J of Inher Metab Disea 36, 139–145. 10.1007/s10545-012-9493-y 22576361

[30] Mathis D , Abela L , Albersen M , Bürer C , Crowther L , Beese K , Hartmann H , Bok LA , Struys E , Papuc SM *et al*. (2016) The value of plasma vitamin B_6_ profiles in early onset epileptic encephalopathies. J Inherit Metab Dis 39, 733–741. 27342130 10.1007/s10545-016-9955-8

[31] Ramos RJ , Albersen M , Vringer E , Bosma M , Zwakenberg S , Zwartkruis F , Jans JJM & Verhoeven‐Duif NM (2019) Discovery of pyridoxal reductase activity as part of human vitamin B6 metabolism. Biochimica et Biophysica Acta (BBA) – General Subjects 1863, 1088–1097. 30928491 10.1016/j.bbagen.2019.03.019

[32] Ciapaite J , Albersen M , Savelberg SMC , Bosma M , Tessadori F , Gerrits J , Lansu N , Zwakenberg S , Bakkers JPW , Zwartkruis FJT *et al*. (2020) Pyridox(am)ine 5′‐phosphate oxidase (PNPO) deficiency in zebrafish results in fatal seizures and metabolic aberrations. Biochimica et Biophysica Acta (BBA) – Molecular Basis of Disease 1866, 165607. 31759955 10.1016/j.bbadis.2019.165607

[33] Nakano M , Morita T , Yamamoto T , Sano H , Ashiuchi M , Masui R , Kuramitsu S & Yagi T (1999) Purification, molecular cloning, and catalytic activity of Schizosaccharomyces pombe pyridoxal reductase. J Biol Chem 274, 23185–23190. 10438489 10.1074/jbc.274.33.23185

[34] Herrero S , González E , Gillikin JW , Vélëz H & Daub ME (2011) Identification and characterization of a pyridoxal reductase involved in the vitamin B6 salvage pathway in Arabidopsis. Plant Mol Biol 76, 157–169. 21533842 10.1007/s11103-011-9777-x

[35] Ito T & Downs DM (2020) Pyridoxal reductase, PdxI, is critical for salvage of pyridoxal in *Escherichia coli* . J Bacteriol 202, 10‐1128. 10.1128/JB.00056-20 PMC7253606 32253339

[36] Tramonti A , Donkor AK , Parroni A , Musayev FN , Barile A , Ghatge MS , Graziani C , Alkhairi M , Al Awadh M , Di Salvo ML *et al*. (2023) Functional and structural properties of pyridoxal reductase (PdxI) from *Escherichia coli* : a pivotal enzyme in the vitamin B6 salvage pathway. FEBS J 290, 5628–5651. 37734924 10.1111/febs.16962 PMC10872706

[37] Mindnich RD & Penning TM (2009) Aldo‐keto reductase (AKR) superfamily: genomics and annotation. Hum Genomics 3, 362. 19706366 10.1186/1479-7364-3-4-362 PMC3206293

[38] Barski OA , Tipparaju SM & Bhatnagar A (2008) The Aldo‐keto reductase superfamily and its role in drug metabolism and detoxification. Drug Metab Rev 40, 553–624. 18949601 10.1080/03602530802431439 PMC2663408

[39] Penning TM , Wangtrakuldee P & Auchus RJ (2019) Structural and functional biology of Aldo‐keto reductase steroid‐transforming enzymes. Endocr Rev 40, 447–475. 30137266 10.1210/er.2018-00089 PMC6405412

[40] O'Connor T , Ireland LS , Harrison DJ & Hayes JD (1999) Major differences exist in the function and tissue‐specific expression of human aflatoxin B1 aldehyde reductase and the principal human aldo‐keto reductase AKR1 family members. PMC1220579 10510318

[41] Zeng C‐M , Chang LL , Ying MD , Cao J , He QJ , Zhu H & Yang B (2017) Aldo–keto reductase AKR1C1–AKR1C4: functions, regulation, and intervention for anti‐cancer therapy. Front Pharmacol 8, 119. 28352233 10.3389/fphar.2017.00119 PMC5349110

[42] Penning TM , Burczynski ME , Jez JM , Hung CF , Lin HK , Ma H , Moore M , Palackal N & Ratnam K (2000) Human 3α‐hydroxysteroid dehydrogenase isoforms (AKR1C1–AKR1C4) of the aldo‐keto reductase superfamily: functional plasticity and tissue distribution reveals roles in the inactivation and formation of male and female sex hormones. Biochemical Journal 351, 67–77. 10998348 10.1042/0264-6021:3510067 PMC1221336

[43] Burczynski ME , Sridhar GR , Palackal NT & Penning TM (2001) The reactive oxygen species‐ and Michael acceptor‐inducible human Aldo‐keto reductase AKR1C1 reduces the α,β‐unsaturated aldehyde 4‐Hydroxy‐2‐nonenal to 1,4‐Dihydroxy‐2‐nonene. J Biol Chem 276, 2890–2897. 11060293 10.1074/jbc.M006655200

[44] Penning TM , Jonnalagadda S , Trippier PC & Rižner TL (2021) Aldo‐keto reductases and cancer drug resistance. Pharmacol Rev 73, 1150–1171. 34312303 10.1124/pharmrev.120.000122 PMC8318518

[45] Yokochi N , Yoshikane Y , Trongpanich Y , Ohnishi K & Yagi T (2004) Molecular cloning, expression, and properties of an unusual Aldo‐keto reductase family enzyme, pyridoxal 4‐dehydrogenase, that catalyzes irreversible oxidation of pyridoxal. J Biol Chem 279, 37377–37384. 15226311 10.1074/jbc.M405344200

[46] Yokochi N , Nishimura S , Yoshikane Y , Ohnishi K & Yagi T (2006) Identification of a new tetrameric pyridoxal 4‐dehydrogenase as the second enzyme in the degradation pathway for pyridoxine in a nitrogen‐fixing symbiotic bacterium, *Mesorhizobium loti* . Arch Biochem Biophys 452, 1–8. 16824480 10.1016/j.abb.2006.06.002

[47] Harruff RC & Jenkins WT (1976) A13 C n.m.r. study of the B6 vitamins and of their aldimine derivatives. Org Magn Reson 8, 548–557.

[48] Schlegel BP , Jez JM & Penning TM (1998) Mutagenesis of 3α‐hydroxysteroid dehydrogenase reveals a “push−pull” mechanism for proton transfer in Aldo−keto reductases. Biochemistry 37, 3538–3548. 9521675 10.1021/bi9723055

[49] Jin Y & Penning TM (2006) Multiple steps determine the overall rate of the reduction of 5α‐dihydrotestosterone catalyzed by human type 3 3α‐hydroxysteroid dehydrogenase: implications for the elimination of androgens. Biochemistry 45, 13054–13063. 17059222 10.1021/bi060591r PMC2597410

[50] Zhao S , Wagn S , Zhao Z & Li W (2019) AKR1C1‐3, notably AKR1C3, are distinct biomarkers for liver cancer diagnosis and prognosis: database mining in malignancies. Oncol Lett 18, 4515–4522. 31611960 10.3892/ol.2019.10802 PMC6781771

[51] Dudkina N , Park HB , Song D , Jain A , Khan SA , Flavell RA , Johnson CH , Palm NW & Crawford JM (2025) Human AKR1C3 binds agonists of GPR84 and participates in an expanded polyamine pathway. Cell Chemical Biology 32, 126–144. 39163853 10.1016/j.chembiol.2024.07.011 PMC11748234

[52] Rižner TL , Šmuc T , Rupreht R , Šinkovec J & Penning TM (2006) AKR1C1 and AKR1C3 may determine progesterone and estrogen ratios in endometrial cancer. Mol Cell Endocrinol 248, 126–135. 16338060 10.1016/j.mce.2005.10.009

[53] Oka T (2001) Modulation of gene expression by vitamin B6. NRR 14, 257. 10.1079/NRR200125 19087426

[54] Fu C , Donovan WP , Shikapwashya‐Hasser O , Ye X & Cole RH (2014) Hot fusion: an efficient method to clone multiple DNA fragments as well as inverted repeats without ligase. PLoS One 9, e115318. 25551825 10.1371/journal.pone.0115318 PMC4281135

[55] Ito T , Ogawa H , Hemmi H , Downs DM & Yoshimura T (2022) Mechanism of pyridoxine 5′‐phosphate accumulation in pyridoxal 5′‐phosphate‐binding protein deficiency. J Bacteriol 204, e00521‐21. 34978460 10.1128/jb.00521-21 PMC8923219

[56] Bugnon M , Röhrig UF , Goullieux M , Perez MAS , Daina A , Michielin O & Zoete V (2024) SwissDock 2024: major enhancements for small‐molecule docking with attracting cavities and AutoDock Vina. Nucleic Acids Res 52, W324–W332. 38686803 10.1093/nar/gkae300 PMC11223881

