# Supplementary material for: A new branch of mammalian vitamin B6 metabolism: AKR1C‐mediated conversion of pyridoxal to pyridoxine and 4‐pyridoxolactone
Source: FEBS J. 2026 Feb 25;293(16):4841–59. doi: 10.1111/febs.70471 (PMC13489509; doi:10.1111/febs.70471)
Supplement: Supplementary file 1 [file FEBS-293-4841-s001.pdf]

Supplementary information for Kito et al.

A New Branch of Mammalian Vitamin B_6 Metabolism: AKR1C-Mediated Conversion of Pyridoxal to Pyridoxine and 4-Pyridoxolactone

Supplementary Figures

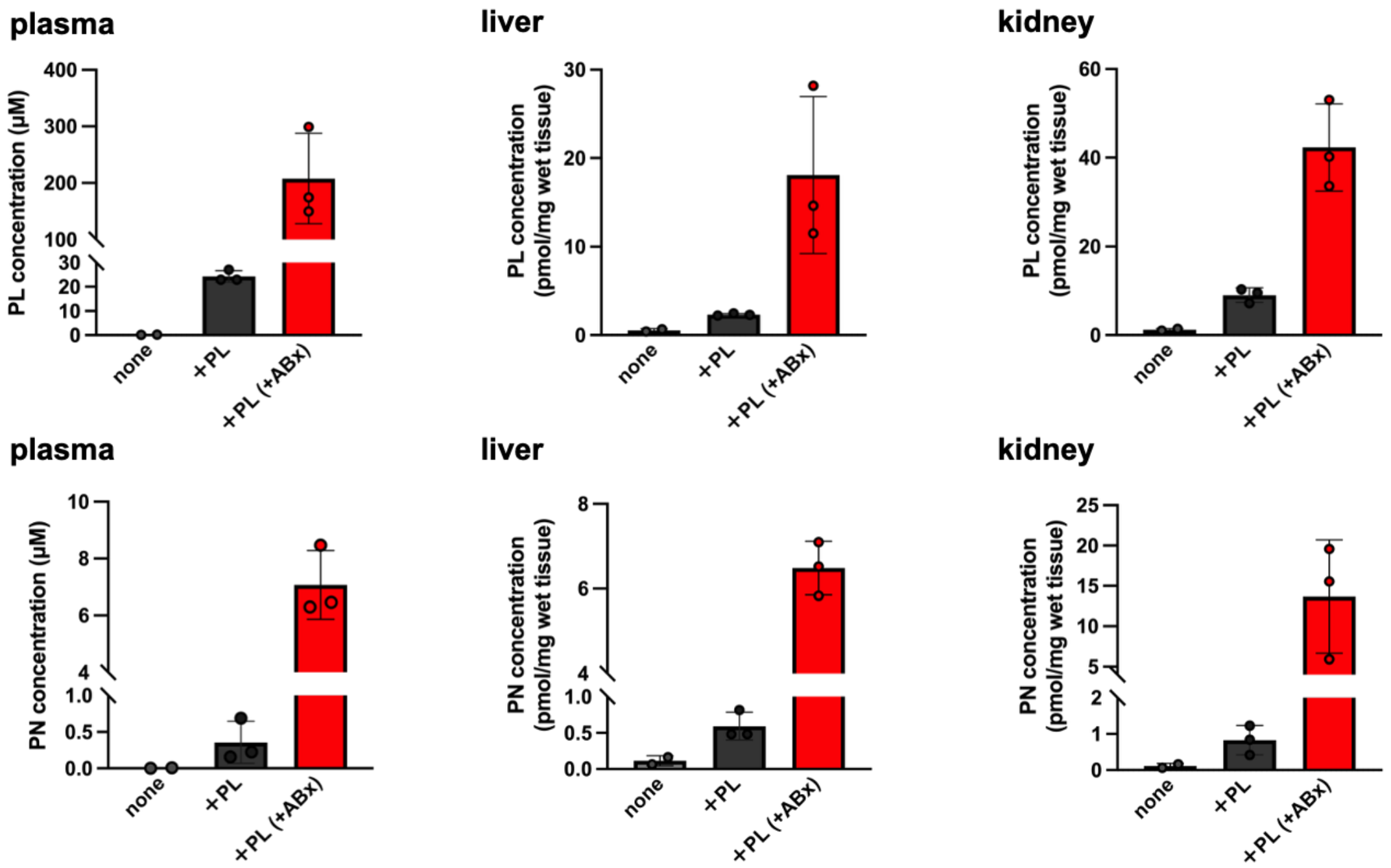

Supplementary Fig. 1

Effect of gut microbiota on PL → PN conversion in mice.

PL or water was orally administered to mice at a dose of 0.4 mL of a 10 mg/mL solution, and plasma, liver, and kidney samples were collected 1 hour after administration. PL concentrations in the plasma, liver, and kidney of PL-treated mice (+PL) were measured by HPLC (upper panels). For reference, PN concentrations in the same tissues following PL administration (+PL) are also shown (bottom panels), corresponding to the data presented in Fig. 2C-E. Data are presented as mean ± SD (n = 3 for PL-administrated mice and n=2 for water-administrated mice).

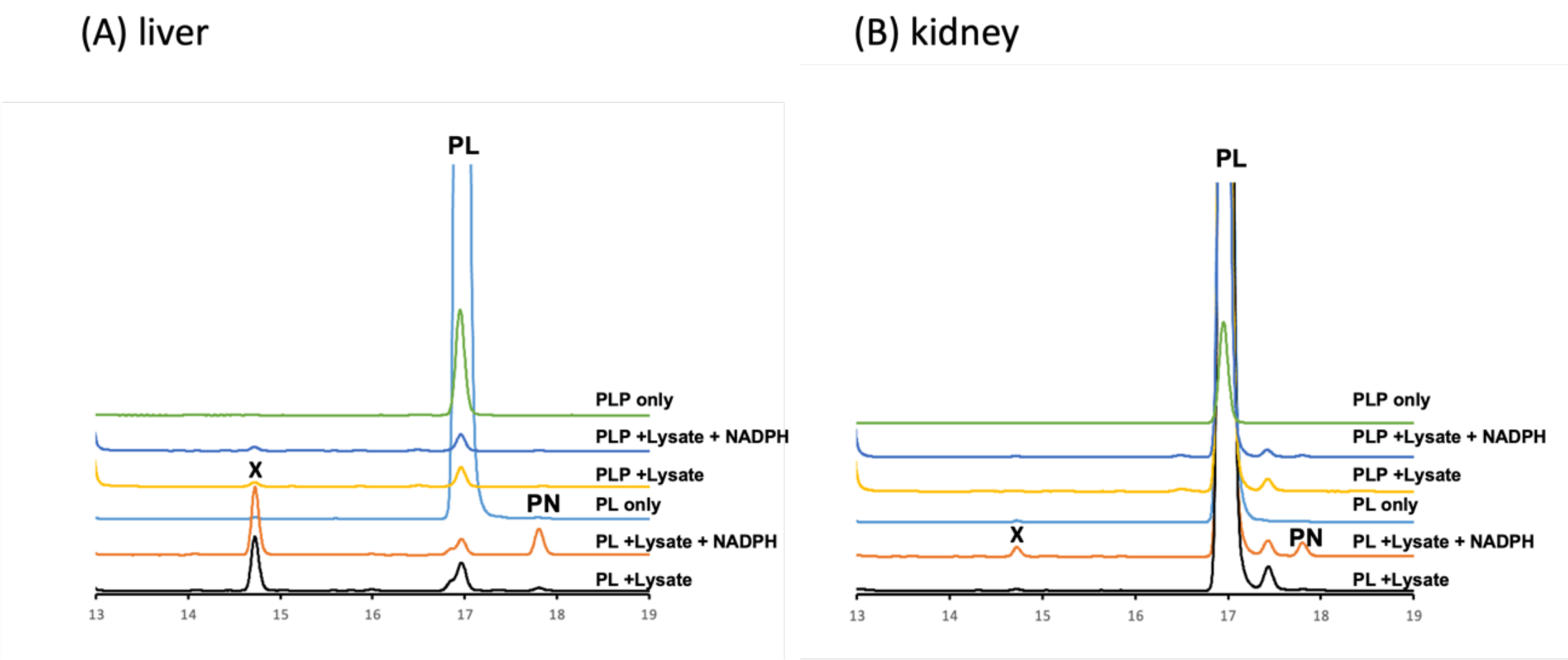

Supplementary Fig. 2

PN production activity in lysate of mouse organs.

PL or PLP was incubated with cell-free extract prepared from mice liver (A) or kidney (B) in the presence or absence of NADPH. PN production was detected only in reactions containing PL and NADPH. Representative HPLC chromatograms of vitamin B_6 analysis are shown. Shown is a representative chromatogram from at least three independent experiments, all of which yielded comparable profiles.

(A)

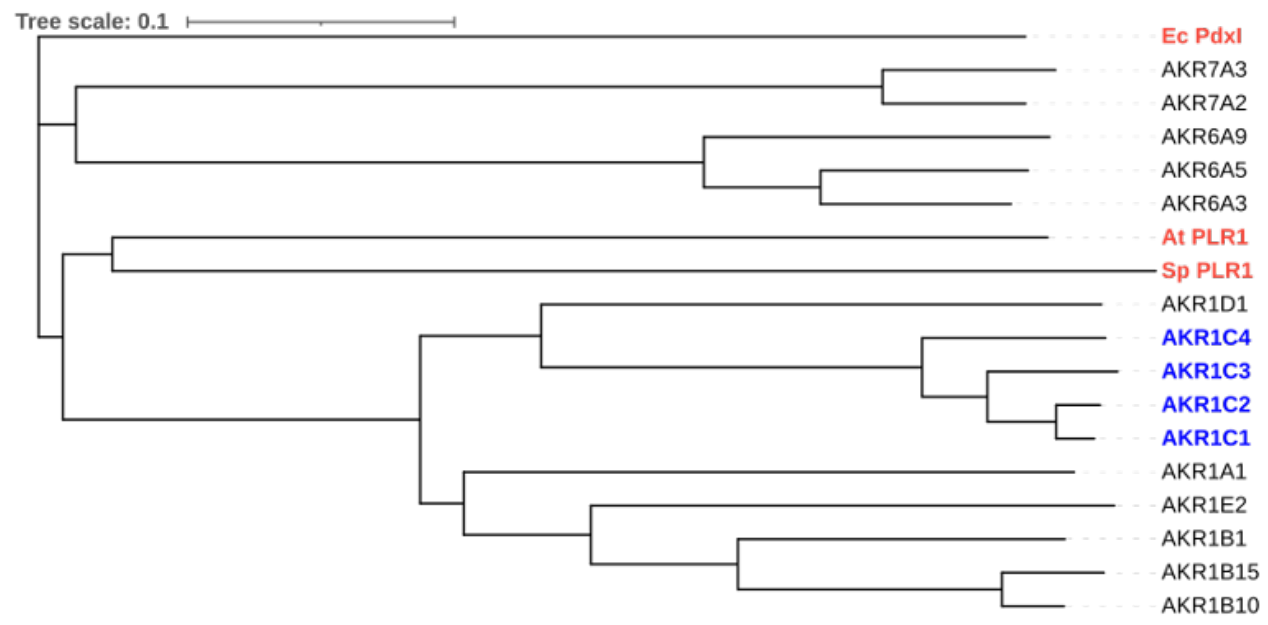

(B)

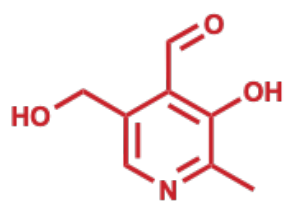

PL

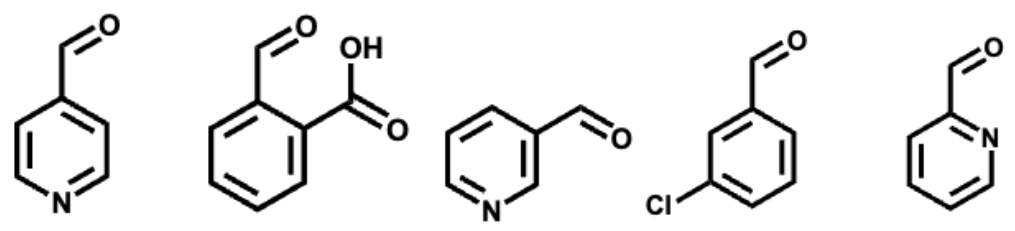

	4PA	2CBA	3PA	3CBA	2PA
AKR1A1	+++	±	+++	+++	++
AKR1B1	++	±	++	++	++
AKR1C1	+	±	±	±	±
AKR1C4	+	±	±	+	±
AKR7A2	++	+++	+	++	+++

Data were from O'Connor T. *et al.* (1999) *Biochemical Journal. 343(2):487-504*

Supplementary Fig. 3

Phylogenetic analysis of human AKR proteins and their reactivity toward PL analogs.

(A) Phylogenetic analysis of human AKR proteins. A phylogenetic tree was constructed using 15 human AKR proteins and known PLRs. Multiple sequence alignment was performed using the ClustalW program (https://www.genome.jp/tools-bin/clustalw) with the following parameters: gap open penalty = 10.0, gap extension penalty = 0.1, and the BLOSUM matrix for proteins was used. The resulting phylogenetic tree was visualized using the Interactive Tree Of Life (iTOL) web server (https://itol.embl.de).

(B) Reactivity of AKR enzymes toward PL analogs. Chemical structures of representative substrates targeted by known human AKR superfamily enzymes and their reported enzyme activities are shown. The table summarizes the specific activity (nmol/min/mg protein) of AKR enzymes toward PL analogs. Activity levels are denoted as follows: +++ >1000, ++ >100, + >10, and ± <10. Data are obtained from Ref. [40] PA: pyridine-4-aldehyde, 2-CBA: 2-carboxybenzaldehyde, 3PA: pyridine-3-aldehyde, 3CBA: 3-chlorobenzaldehyde, 2PA: pyridine-2-aldehyde.

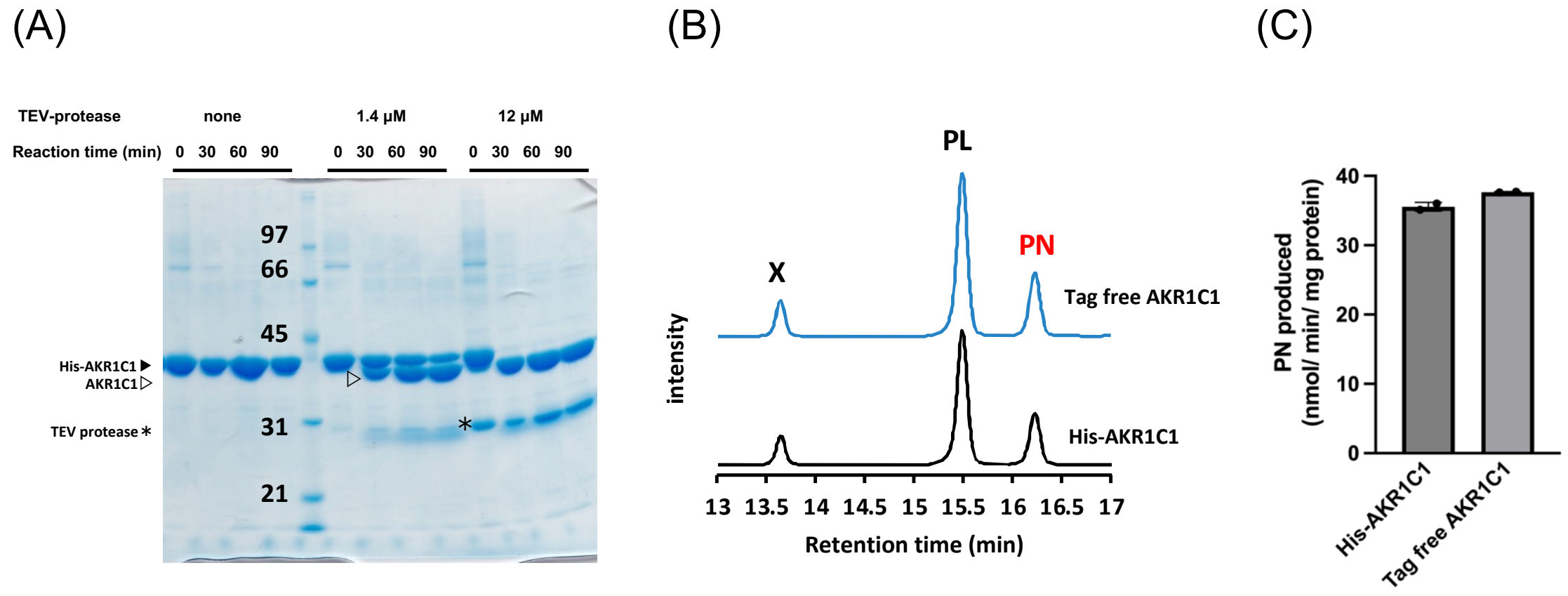

Supplementary Fig. 4

Effect of N-terminal His-tag on the reactivity of AKR1C1 toward PL.

AKR1C1 was treated with TEV protease at a concentration of 0.6 mg/mL in the presence of either 1.4 µM or 12 µM TEV protease at 25 °C for 90 min. Complete removal of the His-tag sequence was achieved with 12 µM TEV protease, and tag-free AKR1C1 was used for subsequent enzymatic activity assays. (B, C) The presence or absence of the His-tag did not affect the production of compound X (4-PLA) or PN. Panels (A) and (B) are the representative results from at least two independent experiments, all of which yielded comparable profiles. Data in (C) represents mean from two independent experiments.

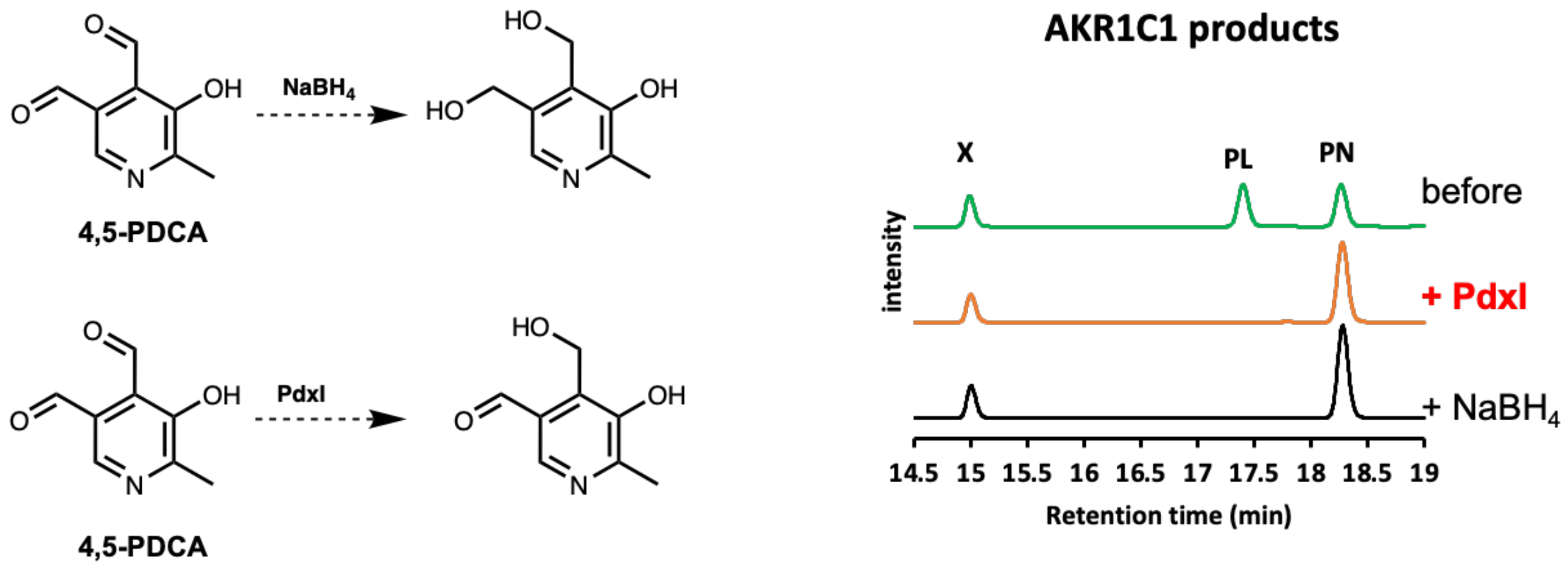

Supplementary Fig. 5

Identification of Compound X.

The reaction product generated by AKR1C1 (compound X) was subjected to treatment with either *E. coli* PL reductase (PdxI) or 10 mM $NaBH_4$ to assess the presence of an aldehyde functional group. PdxI catalyzes hydride transfer to the C4′ aldehyde group of PL. While PdxI readily converted PL present in the reaction mixture to PN, it showed no detectable activity toward compound X, suggesting the absence of an aldehyde group at the C4′ position. Similarly, $NaBH_4$ treatment reduced PL to PN but did not affect compound X. These results further support that compound X lacks a reducible aldehyde group in its structure. The chromatogram is a representative result from at least three independent experiments, all of which yielded comparable profiles.

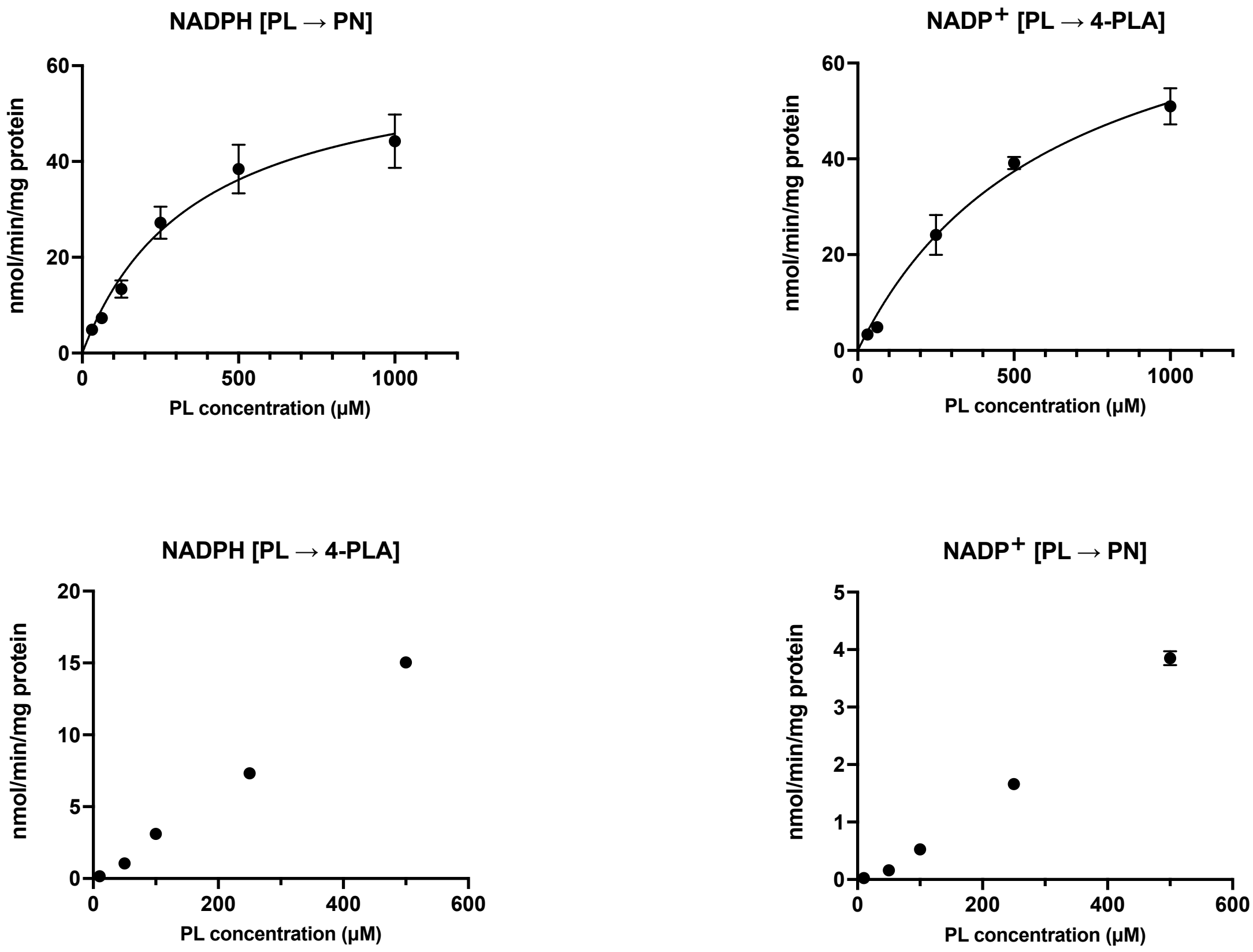

Supplementary Fig. 6

Effect of NADPH and NADP⁺ on AKR1C1-catalyzed PLR and PLD activities.

PLR and PLD activities of AKR1C1 were assayed in the presence of 0.2 mM NADPH or $NADP^+$. PLR activity followed Michaelis–Menten kinetics in the presence of NADPH, whereas PLD activity followed Michaelis–Menten kinetics in the presence of $NADP^+$. For each substrate concentration, initial reaction rates were measured in triplicate and data are presented as mean ± SD. Michaelis–Menten curves were generated by plotting the initial reaction rates against substrate concentrations, and kinetic parameters were obtained by nonlinear regression analysis using GraphPad Prism 10.

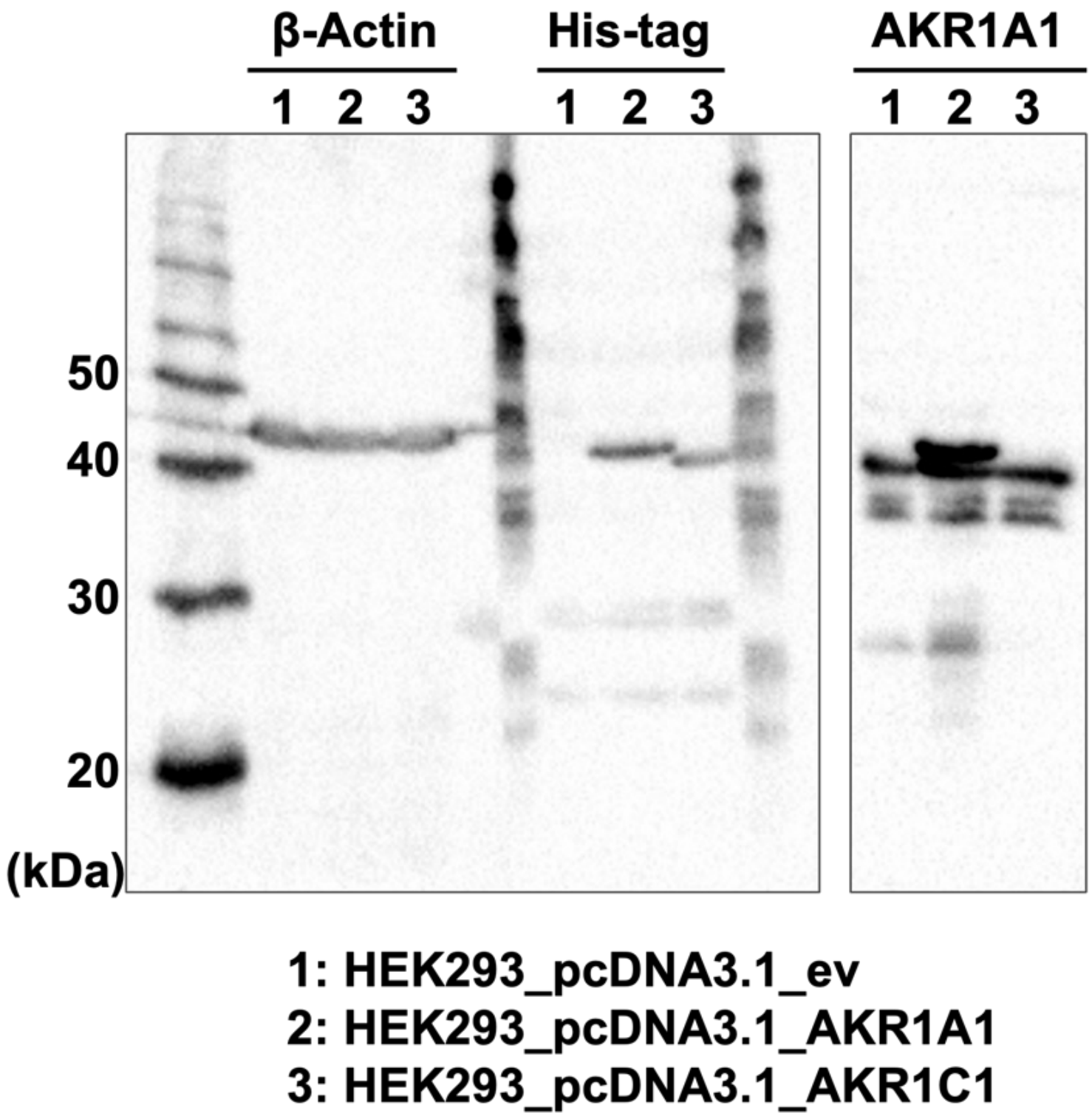

Supplementary Fig. 7

Confirmation of AKR1C1 overexpression in HEK293 cells.

Protein expression in HEK293 cells transfected with expression vectors encoding AKR1C1, AKR1A1, or an empty vector control was examined. Expression of N-terminal His-tagged AKR1C1 and AKR1A1 was confirmed by western blotting analysis using an anti-His-tag antibody. Expression of AKR1A1 was further verified with an AKR1A1-specific antibody. A representative result from at least three independent experiments, all of which yielded comparable profiles, are shown.

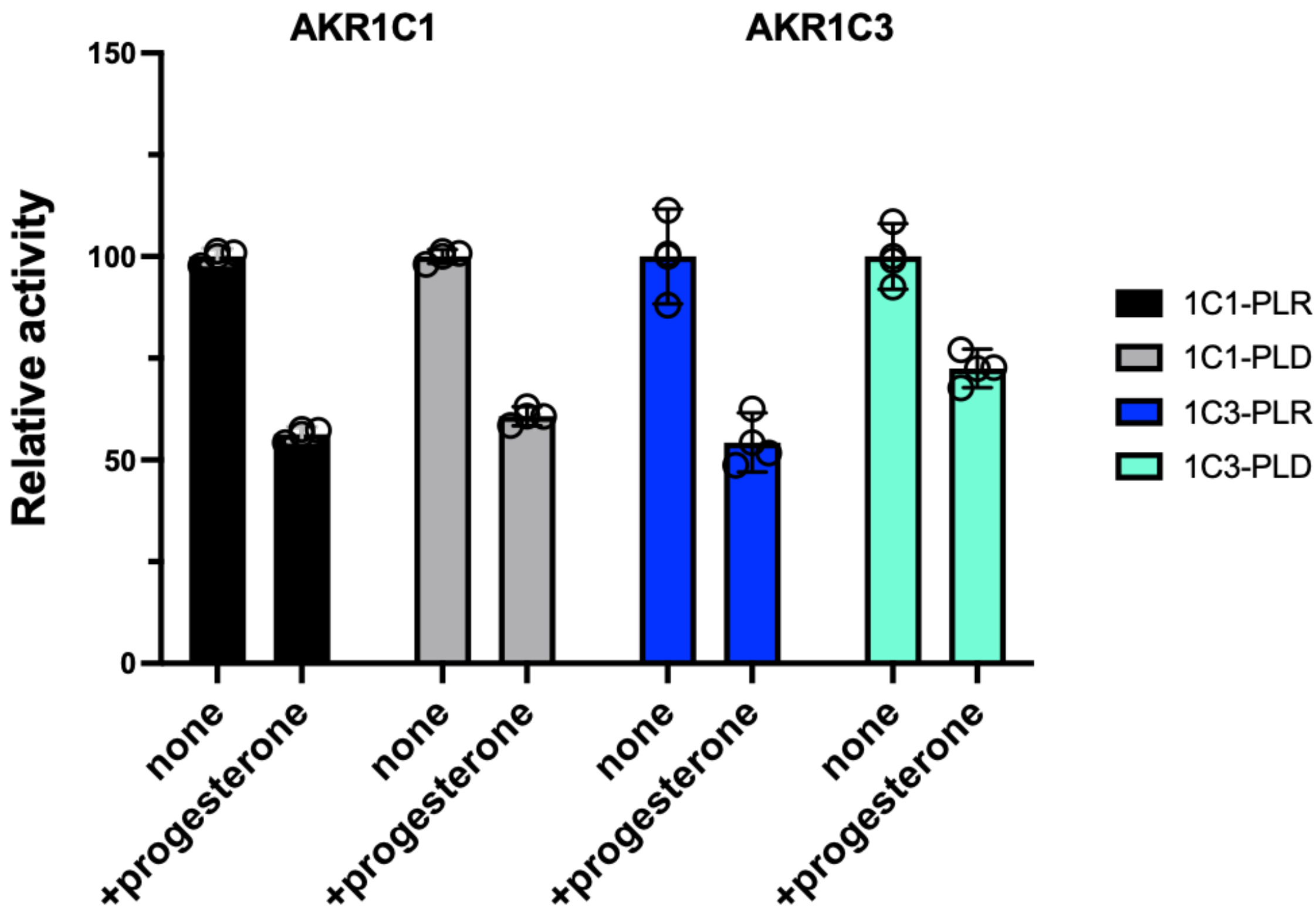

Supplementary Fig. 8

Effect of progesterone on AKR1C1- and AKR1C3-catalyzed PLR and PLD activities.

PLR and PLD activities of AKR1C1 or AKR1C3 were measured in a reaction buffer containing 100 mM sodium phosphate (NaPB) pH 7.0, 0.25 mM PL, and 0.2 mM NADPH, in the absence or presence of 10 μM progesterone. Reactions were carried out at 37 °C for 20 min. The amounts of PN and 4PLA produced were quantified by HPLC. Data are presented as mean ± SD from at least three independent experiments.

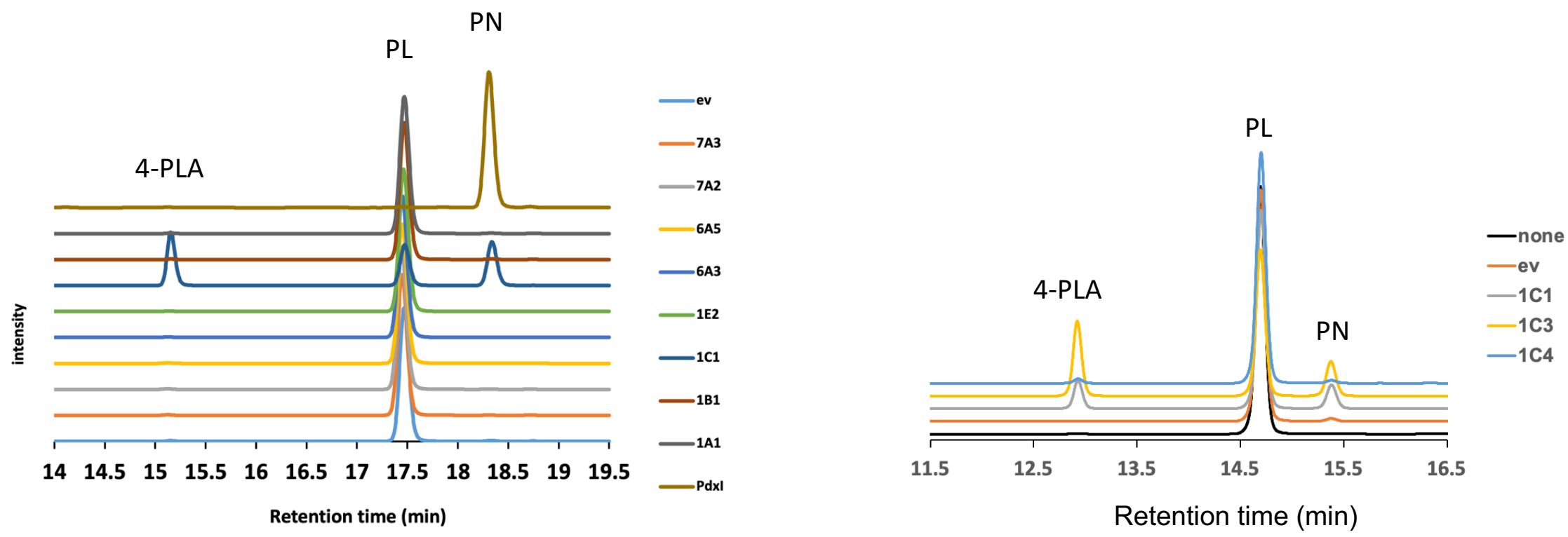

Supplementary Fig. 9

Effect of expression of A**KR isozymes on PL metabolism in *E. coli.***

E. coli BL21 Star^{TM} (DE3) strains harboring pET-TEV vectors encoding various AKR isozymes were precultured in AI-TB medium and subsequently grown in M9-glucose medium supplemented with 0.1 mM PL. As a positive control, BL21 Star^{TM} (DE3) cells expressing *pdxI* from the pCA24N vector were used. After 24 h of cultivation at 30 °C, tculture supernatants were collected and analyzed by HPLC to assess PL-derived metabolites. Shown are representative HPLC chromatogram from at least three independent experiments, all of which yielded comparable profiles.

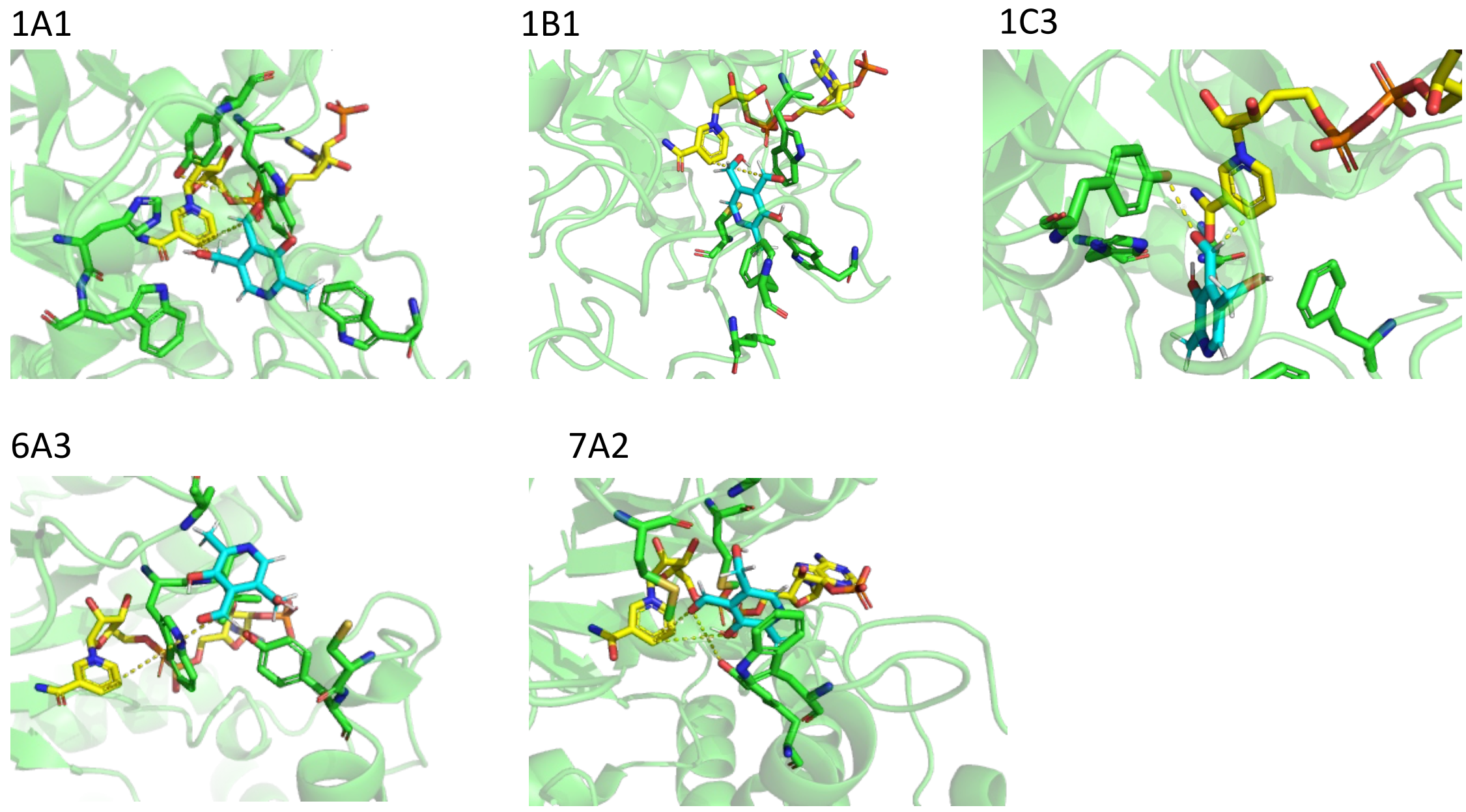

Supplementary Fig. 10

Predicted binding modes of AKR isozymes with PL based on docking simulations.

Docking simulations were performed using SwissDock with the Attracting Cavities 2.0 algorithm (https://swissmodel.expasy.org). Receptor structures used for the simulations included human AKR1C1 (**PDB ID: 6IJX**), AKR1B1 (**1PWM**), AKR1C3 (**1S2C**), and AKR7A2 (**2BP1**), with bound ligands removed prior to docking. For AKR1A1 and AKR6A3, NADP(H)-bound structures were generated using AlphaFold2 models and AlphaFill (https://alphafill.eu). The PL binding site was defined as a cubic region with 20 Å side centered on the C4 atom of the bound NADP(H) cofactor. For each isozyme, the binding pose with the lowest predicted binding free energy was selected and visualized using PyMOL (https://www.pymol.org).

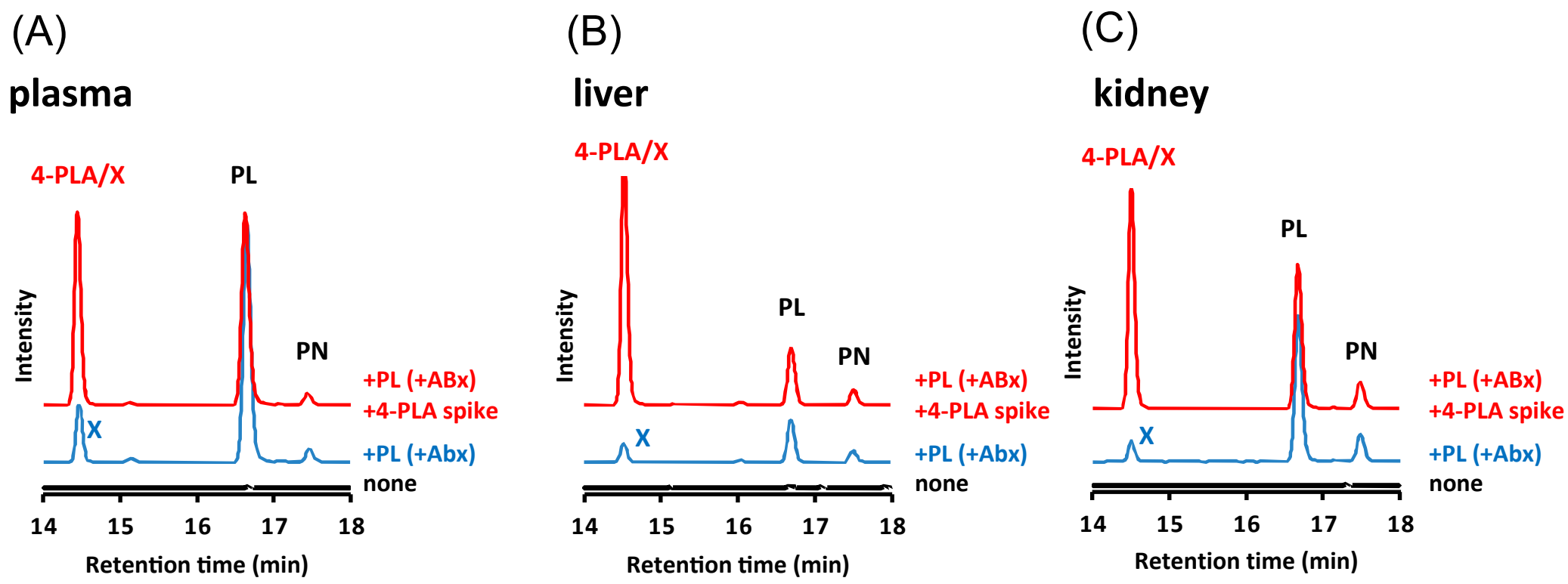

Supplementary Fig. 11

Confirmation of PL-to-4-PLA conversion in mice.

Representative HPLC chromatograms of pooled samples from plasma (A), liver (B), and kidney (C) from antibiotic-treated (ABx) mice administered either water (none) or PL (+PL) are shown.

To confirm the identity of compound X, authentic 4-PLA was added to the samples followed by reanalyzed (+4-PLA spiked). The retention time of compound X matched that of the 4-PLA standard, confirming the formation of 4-PLA from PL in vivo. Shown are representative HPLC chromatogram from at least two independent experiments, all of which yielded comparable profiles.

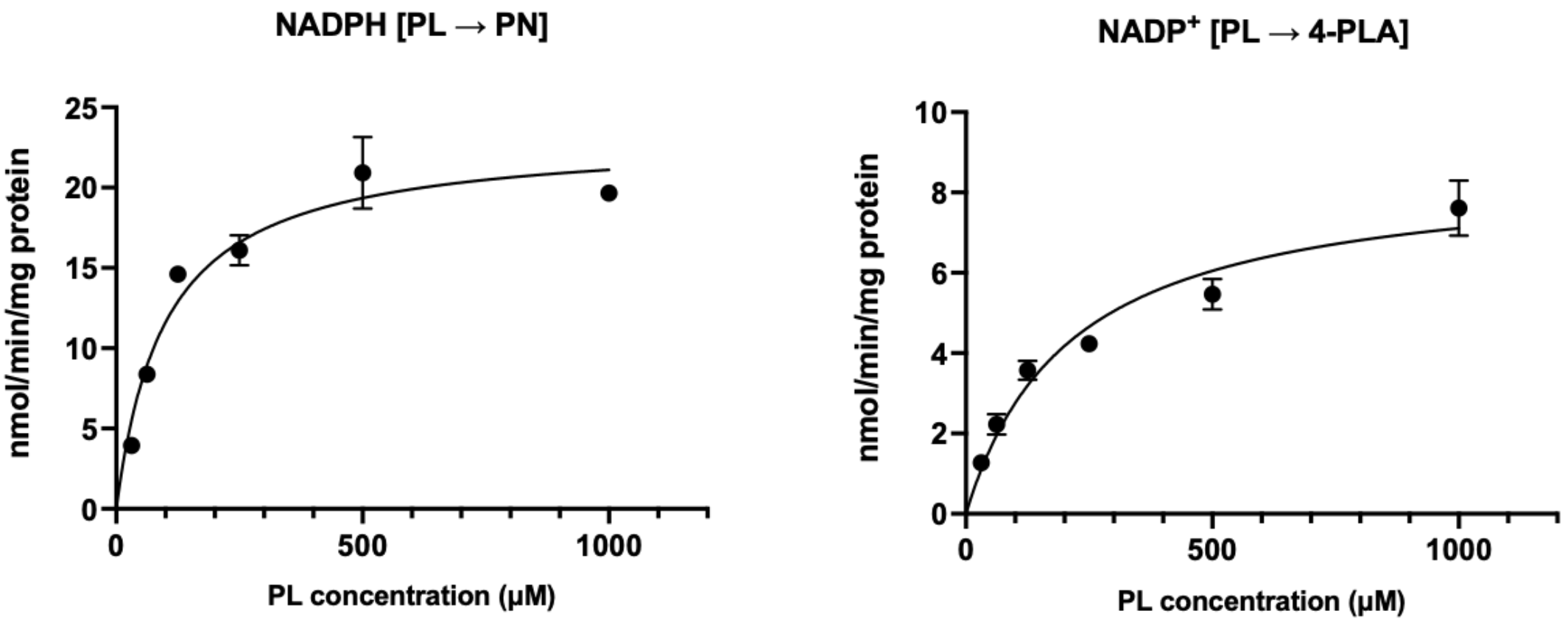

Supplementary Fig. 12

Kinetic parameters of AKR1C6-catalyzed PLR and PLD activities.

The AKR1C6-catalyzed PLR activity (NADPH-dependent PL-to-PN conversion) and PLD activity ($NADP^+$-dependent PL-to-4-PLA conversion) were assayed at 37°C. Reactions were performed in a 200 µL mixture containing 100 mM sodium phosphate buffer (pH 7.0), 0.2 mM NADPH or $NADP^+$, varying concentrations of PL (10~1000 µM), and ~20 µg of purified AKR1C6. For each substrate concentration, initial reaction rates were measured in triplicate and data are presented as mean ± SD. Michaelis–Menten curves were generated by plotting initial reaction rates against substrate concentrations, and kinetic parameters were obtained by nonlinear regression using GraphPad Prism 10.

Nucleotide sequences of AKR isozymes used in this study

>AKR1A1 (**Uniprot: P14550**)

ATGGCAGCAAGCTGTGTTCTGCTGCATACCGGTCAGAAAATGCCGCTGATTG
GTTTAGGCACCTGGAAAAGCGAACCTGGTCAGGTTAAAGCAGCAGTTAAATA
TGCACTGAGCGTTGGTTATCGTCATATTGATTGTGCAGCCATTTATGGCAATGA
ACCGGAAATTGGTGAAGCCCTGAAAGAAGATGTTGGTCCGGGTAAAGCAGT
TCCGCGTGAAGAACTGTTTGTTACCAGCAAACTGTGGAACACCAAACATCAT
CCTGAAGATGTGGAACCGGCACTGCGTAAAACCCTGGCCGATCTGCAACTGG
AATATCTGGATCTGTATCTGATGCATTGGCCGTATGCATTTGAACGTGGTGATA
ATCCGTTTCCGAAAAATGCAGATGGCACCATTTGTTATGATAGCACCCATTATA
AAGAAACCTGGAAAGCACTGGAAGCCCTGGTTGCAAAAGGTCTGGTTCAGG
CACTGGGTCTGAGCAATTTTAACAGCCGTCAGATTGATGATATTCTGAGCGTG
GCAAGCGTTCGTCCGGCAGTTCTGCAAGTTGAATGTCATCCGTATCTGGCAC
AGAATGAACTGATTGCACATTGTCAGGCACGTGGTCTGGAAGTTACCGCATA
TAGTCCGCTGGGTAGCAGCGATCGTGCATGGCGTGATCCGGATGAACCGGTG
CTGCTGGAAGAACCGGTTGTTCTGGCACTGGCAGAAAAATATGGTCGTAGTC
CGGCACAGATTCTGCTGCGTTGGCAGGTTCAGCGTAAAGTTATTTGTATTCCG
AAAAGCATTACCCCGAGCCGTATTCTGCAAAACATTAAAGTGTTTGATTTTAC
CTTTAGTCCGGAAGAGATGAAACAGCTGAATGCCCTGAATAAAAACTGGCGT
TATATTGTTCCGATGCTGACCGTTGATGGTAAACGTGTTCCTCGTGATGCAGG
TCATCCGCTGTATCCGTTTAATGATCCGTATTAA

>AKR1B1 (**Uniprot: P15121**)

ATGGCAAGCCGTCTGCTGCTGAATAATGGTGCAAAAATGCCGATTTTAGGTCT
TGGCACCTGGAAAAGTCCGCCTGGTCAGGTTACCGAAGCAGTTAAAGTTGC
AATTGATGTGGGTTATCGCCATATTGATTGTGCCCATGTTTATCAGAATGAAAA
CGAAGTTGGTGTGGCCATCCAAGAAAAACTGCGTGAACAGGTTGTTAAACG
CGAAGAACTGTTTATTGTTAGCAAACTGTGGTGCACCTATCATGAAAAAGGT
CTGGTTAAAGGTGCATGTCAGAAAACCCTGAGCGATCTGAAACTGGATTATC
TGGATCTGTATCTGATTCATTGGCCGACCGGCTTTAAACCGGGTAAAGAATTT
TTTCCGCTGGATGAAAGCGGTAATGTTGTTCCGAGCGATACCAATATTCTGGA
TACCTGGGCAGCAATGGAAGAACTGGTTGATGAAGGCCTGGTGAAAGCAAT
TGGTATCAGCAATTTTAACCACCTTCAGGTGGAAATGATTCTGAACAAACCG
GGTCTGAAATACAAACCGGCAGTTAATCAGATTGAATGCCATCCGTATCTGAC
CCAAGAGAAACTGATTCAGTATTGTCAGAGCAAAGGTATTGTTGTGACCGCA

TATAGTCCGCTGGGTAGTCCGGATCGTCCGTGGGCAAAACCGGAAGATCCGA
GCCTGCTGGAAGATCCTCGTATTAAAGCCATTGCAGCCAAACATAACAAAAC
CACCGCACAGGTTCTGATTCGTTTTCCGATGCAGCGTAATCTGGTTGTTATTC
CGAAAAGCGTTACACCGGAACGTATTGCCGAAAACTTCAAAGTGTTTGATTT
TGAACTGAGCAGCCAGGATATGACCACACTGCTGAGCTATAATCGTAATTGGC
GTGTTTGTGCACTGCTGTCATGTACCAGCCATAAAGATTATCCGTTTCACGAA
GAGTTTTAA

>AKR1C1 (**Uniprot: Q04828**)
ATGGATAGCAAATATCAGTGCGTGAAACTGAACGATGGTCATTTTATGCCGGT
TTTAGGTTTTGGCACCTATGCACCGGCAGAAGTTCCGAAAAGCAAAGCACTG
GAAGCAACCAAACTGGCAATTGAAGCAGGTTTTCGTCATATTGATAGCGCAC
ATCTGTATAACAACGAAGAACAGGTTGGTCTGGCAATTCGTAGCAAAATTGC
AGATGGTAGCGTTAAACGCGAGGATATCTTTTATACCAGCAAACTGTGGTGTA
ATAGCCATCGTCCGGAACTGGTTCGTCCTGCACTGGAACGTAGCCTGAAAAA
TCTGCAGCTGGATTATGTTGATCTGTATCTGATTCATTTTCCGGTTAGCGTGAA
ACCGGGTGAAGAGGTTATTCCGAAAGATGAAAATGGCAAAATCCTGTTCGAT
ACCGTGGATCTGTGTGCAACCTGGGAAGCAGTTGAAAAATGTAAAGATGCA
GGTCTGGCCAAAAGCATTGGTGTTAGCAATTTTAACCGTCGTCAGCTGGAAA
TGATTCTGAATAAACCGGGTCTGAAATACAAACCGGTGTGTAATCAGGTTGA
ATGCCATCCGTATTTTAACCAGCGTAAACTGCTGGATTTCTGCAAATCCAAAG
ATATTGTTCTGGTTGCATATAGCGCACTGGGTAGTCATCGTGAAGAACCGTGG
GTTGATCCGAATAGTCCGGTTCTGCTGGAAGATCCGGTGCTGTGTGCACTGG
CAAAGAAACATAAACGTACACCGGCACTGATTGCACTGCGTTATCAGCTGCA
GCGTGGTGTTGTTGTTCTGGCAAAAAGCTATAATGAACAGCGTATTCGTCAG
AACGTTCAGGTGTTTGAATTTCAGCTGACCAGCGAAGAAATGAAAGCAATTG
ATGGTCTGAATCGCAATGTTCGTTATCTGACCCTGGATATTTTTGCAGGTCCGC
CTAATTATCCGTTTAGCGACGAATATTAA

>AKR1C3 (**Uniprot: P42330**)
ATGGATTCAAAGCACCAATGTTTAAAACTAAACGATGGCCATTTTATGCCGGT
TCTCGGTTTTGGCACGTACGCTCCGCCTGAGGTGCCGCGTAGCAAGGCCCTT
GAGGTTACCAAATTGGCGATTGAGGCCGGCTTCCGCCATATTGACTCGGCGC
ACCTGTATAATAACGAGGAACAAGTTGGTCTCGCGATCCGTTCCAAAATCGC
AGATGGAAGCGTTAAAAGAGAAGATATCTTCTACACCAGCAAGCTGTGGTCC
ACCTTTCATCGTCCGGAGTTGGTTCGCCCGGCGCTGGAGAACTCCCTCAAAA

AAGCTCAGTTGGATTATGTTGACCTGTACCTGATCCACAGCCCGATGTCGCTA
AAGCCAGGTGAAGAGCTGTCGCCGACCGATGAAAACGGCAAGGTTATTTC
GATATCGTGGACCTGTGCACGACCTGGGAAGCAATGGAAAAGTGCAAGGAC
GCAGGTTTGGCAAAGTCTATTGGCGTATCTAATTTCAACCGTCGTCAATTAGA
GATGATCCTGAATAAACCGGGTTTAAAGTACAAGCCGGTGTGTAATCAGGTG
GAATGTCACCCGTACTTCAACCGCAGCAAACTGCTGGACTTCTGCAAAAGCA
AGGACATCGTGTTGGTGGCGTATTCCGCGCTGGGTAGCCAACGTGATAAACG
TTGGGTTGACCCGAATAGCCCGGTCCTGCTGGAGGATCCGGTGCTGTGCGCG
CTGGCGAAGAAACATAAACGCACCCCGGCGCTGATTGCGTTGCGGTACCAAC
TGCAGCGTGGTGTTGTGGTGCTGGCCAAAAGCTATAACGAGCAACGTATTCG
TCAGAATGTCCAGGTATTTGAATTCCAGCTGACTGCTGAAGATATGAAGGCC
ATTGACGGCTTGGACCGCAACCTGCACTACTTTAACAGCGATTCTTTTGCTAG
CCACCCAAACTATCCGTACAGCGACGAATATTAA

>AKR1C4 (**Uniprot: P17516**)
ATGGATCCCAAATATCAAAGGGTAGAACTAAACGATGGTCATTTTATGCCGGT
ACTGGGTTTTGGTACGTACGCACCGCCTGAAGTTCCGCGTAATCGCGCAGTC
GAGGTTACCAAACTAGCGATTGAAGCTGGCTTCCGCCACATCGACAGCGCGT
ATCTGTACAACAACGAGGAACAAGTGGGTCTCGCCATCCGCAGCAAGATCGC
CGATGGCAGCGTTAAACGTGAGGACATCTTCTATACCAGCAAACTGTGGTGC
ACGTTCTTCCAGCCGCAGATGGTGCAACCGGCGCTTGAGTCGTCCCTGAAAA
AGTTGCAACTGGACTACGTGGACCTGTACCTGCTGCACTTTCCGATGGCATT
GAAGCCAGGTGAAACCCCGCTGCCGAAAGACGAGAACGGTAAAGTGATCTT
CGATACCGTTGATCTGTCAGCCACCTGGGAAGTGATGGAAAAATGCAAAGAT
GCGGGTCTGGCGAAGTCTATTGGCGTGAGCAATTTTAACTGCCGTCAGCTGG
AGATGATTCTGAATAAGCCGGGCCTTAAGTACAAACCAGTCTGTAACCAAGT
AGAGTGCCATCCGTATCTGAATCAGTCCAAGCTGTTGGACTTCTGCAAGTCC
AAGGACATCGTGTTGGTTGCACATAGCGCGCTCGGCACCCAGCGTCACAAGC
TGTGGGTTGACCCGAATAGCCCGGTTCTGTTGGAGGATCCGGTGCTGTGTGC
GTTGGCGAAGAAGCACAAACAGACTCCGGCTTTAATTGCGCTGCGTTACCAG
CTGCAACGTGGTGTTGTGGTGTTAGCTAAATCCTATAACGAGCAGCGTATCCG
CGAGAACATTCAGGTTTTTGAATTCCAACTGACCTCTGAAGATATGAAAGTCT
TGGACGGCCTGAACAGAAACTATCGTTACGTTGTGATGGATTTCCTGATGGA
CCACCCGGATTACCCGTTTAGCGATGAATATTAA

>AKR1D1 (**Uniprot: P51857**)

ATGGATCTATCAGCAGCTAGTCACAGGATACCACTGAGCGATGGTAATAGCAT
TCCAATTATCGGCTTAGGCACGTATAGCGAACCGAAGTCGACCCCGAAGGGC
GCGTGTGCTACGTCCGTGAAAGTCGCCATCGACACCGGTTATCGTCACATCG
ACGGCGCGTACATCTACCAAAACGAGCACGAAGTTGGTGAAGCGATTCGTG
AGAAGATCGCGGAAGGTAAAGTACGCAGAGAAGATATTTTCTATTGTGGTAA
ACTGTGGGCAACCAATCATGTTCCGGAAATGGTGCGTCCGACCTTGGAGCGC
ACCCTGCGTGTTCTTCAGCTGGATTATGTTGATCTGTATATCATCGAGGTGCCG
ATGGCATTCAAACCGGGTGATGAGATCTACCCGCGTGATGAGAACGGCAAGT
GGCTGTACCATAAGTCTAACCTTTGCGCGACCTGGGAAGCCATGGAAGCGTG
CAAAGACGCTGGTTTGGTTAAATCGCTGGGCGTGTCCAACTTTAATCGTCGC
CAGCTGGAGCTGATTCTGAACAAGCCGGGTCTAAAGCACAAACCGGTCAGC
AACCAGGTTGAGTGCCATCCGTACTTCACCCAGCCGAAGCTGCTTAAGTTCT
GCCAACAACACGACATCGTGATTACCGCATACAGCCCGCTGGGAACGAGCCG
TAATCCGATTTGGGTGAACGTGTCTAGCCCTCCGCTGTTGAAGGACGCGCTG
TTGAATTCCCTGGGCAAACGTTATAACAAAACTGCGGCACAGATCGTGCTGC
GCTTCAACATTCAACGTGGTGTGGTTGTCATTCCGAAGTCTTTTAACCTGGAG
CGCATCAAAGAAAACTTCCAGATTTTTGACTTTAGCTTGACCGAAGAGGAAA
TGAAAGACATCGAGGCTCTGAATAAGAACGTTCGTTTTGTTGAGCTGTTAAT
GTGGCGTGACCACCCAGAATACCCGTTTCATGATGAGTACTAA

>AKR1E2 (**Uniprot: Q96JD6**)
ATGGGTGATATTCCGGCAGTTGGTCTGAGCAGCTGGAAAGCAAGTCCGGGTA
AAGTTACCGAAGCAGTTAAAGAAGCAATTGACGCAGGTTATCGTCATTTTGA
TTGCGCCTATTTCTATCACAATGAACGTGAAGTTGGTGCCGGTATTCGTTGCA
AAATCAAAGAAGGTGCAGTTCGTCGCGAGGACCTGTTTATTGCAACCAAACT
GTGGTGTACCTGTCACAAAAAAAGCCTGGTTGAAACCGCATGTCGTAAAAG
CCTGAAAGCACTGAAACTGAATTATCTGGATCTGTATCTGATTCATTGGCCGA
TGGGTTTTAAACCGCCTCATCCGGAATGGATTATGAGCTGTAGCGAACTGAGC
TTTTGTCTGAGCCATCCGCGTGTTCAGGATCTGCCGCTGGATGAAAGCAATAT
GGTTATTCCGAGCGATACCGATTTTCTGGATACCTGGGAAGCAATGGAAGATC
TGGTTATTACCGGTCTGGTTAAAAACATTGGTGTGAGCAACTTTAACCACGA
ACAGCTGGAACGTCTGCTGAATAAACCGGGTCTGCGCTTTAAACCTCTGACC
AATCAGATTGAATGTCATCCGTATCTGACCCAGAAAAACCTGATTAGCTTTTG
CCAGAGCCGTGATGTTAGCGTTACCGCATATCGTCCGTTAGGTGGTAGCTGTG
AAGGTGTTGATCTGATTGATAATCCGGTGATTAAACGCATTGCCAAAGAACAT
GGTAAAAGTCCGGCACAGATTCTGATTCGTTTTCAGATTCAGCGTAACGTGAT

TGTTATTCCGGGTAGCATTACCCCGAGCCATATTAAAGAAAACATCCAGGTGT
TTGATTTCGAACTGACCCAGCATGATATGGATAACATTCTGAGCCTGAATCGT
AATCTGCGTCTGGCAATGTTTCCGATTACCAAAAACCATAAAGATTACCCGTT
CCACATCGAATATTAA

>AKR6A3 (**Uniprot: Q14722**)
ATGCAGGTTAGCATTGCATGCACCGAACACAATCTGAAAAGCCGTAATGGTG
AAGATCGTCTGCTGAGCAAACAGAGCAGCACCGCACCGAATGTTGTTAATGC
AGCACGTGCAAAATTTCGTACCGTTGCAATTATTGCACGTAGCCTGGGCACCT
TTACACCGCAGCATCATATTAGCCTGAAAGAAAGCACCGCAAAACAGACCGG
TATGAAATATCGTAATCTGGGTAAAAGCGGTCTGCGTGTTAGCTGTTTAGGTT
TAGGCACCTGGGTTACCTTTGGTGGTCAGATTAGTGATGAAGTTGCAGAACG
TCTGATGACCATTGCATATGAAAGCGGTGTTAACCTGTTTGATACAGCCGAAG
TTTATGCAGCAGGTAAAGCAGAAGTTATTCTGGGCAGCATTATCAAAAAGAA
AGGTTGGCGTCGTAGCAGCCTGGTTATTACCACCAAACTGTATTGGGGTGGTA
AAGCCGAAACCGAACGTGGTCTGAGCCGTAAACATATTATTGAAGGTCTGAA
AGGTAGCCTGCAGCGTCTGCAGCTGGAATATGTTGATGTTGTTTTTGCAAATC
GTCCGGATAGCAATACCCCGATGGAAGAAATTGTTCGTGCAATGACCCATGT
GATTAACCAAGGTATGGCAATGTACTGGGGCACCAGCCGTTGGAGCGCAATG
GAAATTATGGAAGCATATAGCGTTGCCCGTCAGTTTAATATGATTCCGCCTGTT
TGTGAACAGGCAGAATATCACCTGTTTCAGCGTGAAAAAGTTGAAGTTCAGC
TGCCGGAACTGTATCATAAAATTGGTGTTGGTGCCATGACCTGGTCACCGCTG
GCATGTGGTATTATTAGCGGTAAATATGGTAATGGTGTTCCGGAAAGCAGCCG
TGCAAGCCTGAAATGTTATCAGTGGCTGAAAGAACGTATTGTGAGCGAAGAA
GGTCGTAAACAGCAGAACAAACTGAAAGATCTGAGCCCGATTGCCGAACGC
CTGGGTTGTACCCTGCCGCAGCTGGCAGTTGCATGGTGTCTGCGTAATGAAG
GTGTTAGCAGCGTTCTGCTGGGTAGCAGCACACCGGAACAGCTGATTGAAA
ATCTGGGTGCAATTCAGGTTCTGCCGAAAATGACCAGCCATGTTGTGAATGA
AATTGATAATATCCTGCGCAACAAACCGTACAGCAAAAAAGATTATCGTAGCT
AA

>AKR6A5 (**Uniprot: Q13303**)
ATGTATCCGGAAAGCACCACCGGTAGTCCGGCACGTCTGAGCCTGCGTCAGA
CCGGTTCACCGGGTATGATTTATCGTAATCTGGGTAAAAGCGGTCTGCGTGTT
AGCTGTTTAGGTTTAGGCACCTGGGTTACCTTTGGTGGTCAGATTACCGATGA
AATGGCAGAACAGCTGATGACCCTGGCATATGATAATGGTATTAACCTGTTTG

ATACCGCAGAGGTTTATGCAGCAGGTAAAGCAGAAGTTGTTCTGGGCAACAT
CATTAAAAAGAAAGGTTGGCGTCGTAGCAGCCTGGTTATTACCACCAAAATC
TTTTGGGGTGGTAAAGCCGAAACCGAACGTGGTCTGAGCCGTAAACATATTA
TTGAAGGTCTGAAAGCAAGCCTGGAACGTCTGCAGCTGGAATATGTTGATGT
TGTTTTTGCAAATCGCCCTGATCCGAATACACCGATGGAAGAAACCGTTCGT
GCAATGACCCATGTTATTAATCAAGGTATGGCAATGTATTGGGGCACCAGCCG
TTGGAGCAGCATGGAAATTATGGAAGCATATAGCGTTGCCCGTCAGTTTAATC
TGACACCGCCTATTTGTGAACAGGCCGAATATCATATGTTTCAGCGTGAAAAA
GTTGAAGTTCAGCTGCCGGAACTGTTTCATAAAATTGGTGTTGGTGCCATGA
CCTGGTCACCGCTGGCATGTGGTATTGTTAGCGGTAAATATGATAGCGGTATTC
CGCCTTATAGCCGTGCAAGCCTGAAAGGTTATCAGTGGCTGAAAGATAAAAT
CCTGAGCGAAGAAGGTCGTCGTCAGCAGGCAAAACTGAAAGAACTGCAGG
CAATTGCAGAACGTCTGGGTTGTACCCTGCCGCAGCTGGCCATTGCATGGTG
TCTGCGTAATGAAGGTGTTAGCAGCGTTCTGCTGGGTGCAAGCAATGCAGAT
CAACTGATGGAAAATATTGGTGCGATTCAGGTTCTGCCGAAACTGAGCAGCA
GCATTATTCATGAAATTGATAGCATCCTGGGGAACAAACCGTATAGCAAAAAA
GATTATCGCAGCTAA

>AKR7A2 (**Uniprot: O43488**)
ATGCTGAGCGCAGCAAGCCGTGTTGTTAGCCGTGCAGCAGTTCATTGTGCAC
TGCGTAGTCCGCCTCCGGAAGCACGTGCACTGGCAATGAGCCGTCCGCCACC
GCCTCGTGTTGCAAGCGTTCTGGGTACAATGGAAATGGGTCGTCGTATGGAT
GCACCGGCAAGCGCAGCCGCAGTTCGTGCATTTCTGGAACGTGGTCATACCG
AACTGGATACCGCATTTATGTATAGTGATGGTCAGAGCGAAACCATTTTAGGT
GGTTTAGGCTTAGGTTTAGGTGGCGGTGATTGTCGTGTTAAAATTGCAACCAA
AGCAAATCCGTGGGATGGTAAAAGCCTGAAACCGGATAGCGTTCGTAGCCAG
CTGGAAACCAGTCTGAAACGTCTGCAATGTCCGCAGGTTGACCTGTTTTATC
TGCATGCACCGGATCATGGTACACCGGTTGAAGAAACCCTGCATGCATGTCA
GCGTCTGCATCAAGAAGGTAAATTTGTTGAACTGGGCCTGAGCAATTATGCA
AGCTGGGAAGTTGCAGAAATTTGTACCCTGTGTAAAAGCAATGGTTGGATTC
TGCCGACCGTTTATCAGGGTATGTATAATGCAACCACACGTCAGGTTGAAACC
GAACTGTTTCCGTGTCTGCGTCATTTTGGTCTGCGTTTTTATGCATATAATCCG
CTGGCAGGCGGTCTGCTGACCGGTAAATACAAATATGAAGATAAAGATGGCA
AACAGCCGGTGGGTCGTTTTTTTGGTAATAGCTGGGCAGAAACCTATCGTAAT
CGTTTTTGGAAAGAACACCACTTTGAAGCAATTGCCCTGGTTGAAAAAGCAC
TGCAAGCAGCCTATGGTGCAAGCGCACCGAGCGTTACCAGCGCAGCACTGC

GTTGGATGTATCATCATAGTCAGCTGCAAGGTGCACATGGTGATGCAGTTATT
CTGGGTATGAGCAGCCTGGAACAGCTGGAACAAAATCTGGCAGCAACCGAA
GAAGGTCCGCTGGAACCGGCAGTTGTTGATGCATTTAATCAGGCATGGCATC
TGGTTGCACATGAATGTCCGAACTATTTTCGCTAA

>AKR7A3 (**Uniprot: O95154**)
ATGAGCCGTCAGCTGAGCCGTGCACGTCCGGCAACCGTTCTGGGTGCAATGG
AAATGGGTCGTCGTATGGATGCACCGACCAGCGCAGCAGTTACCCGTGCATT
TCTGGAACGTGGTCATACCGAAATTGATACCGCATTTGTTTATAGCGAAGGTC
AGAGCGAAACCATTTTAGGTGGTCTGGGTCTGCGCTTAGGTGGTAGCGATTG
TCGTGTTAAAATCGATACCAAAGCAATTCCGCTGTTTGGCAATAGCCTGAAAC
CGGATAGCCTGCGTTTTCAGCTGGAAACCAGTCTGAAACGTCTGCAGTGTCC
GCGTGTTGACCTGTTTTATCTGCATATGCCGGATCATAGCACACCGGTTGAAG
AAACCCTGCGTGCATGTCATCAGCTGCATCAAGAAGGTAAATTTGTTGAACT
GGGCCTGAGCAATTATGCAGCATGGGAAGTTGCAGAAATTTGTACCCTGTGT
AAAAGCAATGGTTGGATTCTGCCGACCGTTTATCAGGGTATGTATAATGCAAT
TACCCGTCAGGTTGAAACCGAACTGTTTCCGTGTCTGCGTCATTTTGGTCTGC
GTTTTTATGCATTTAATCCGCTGGCAGGCGGTCTGCTGACCGGTAAATACAAA
TATGAGGATAAAAATGGCAAACAGCCGGTGGGTCGTTTTTTTGGTAATACCTG
GGCAGAAATGTATCGCAATCGTTATTGGAAAGAACACCACTTTGAAGGTATT
GCCCTGGTTGAAAAAGCACTGCAGGCAGCATATGGTGCAAGCGCACCGAGC
ATGACCAGCGCGACCCTGCGTTGGATGTATCATCATAGCCAGCTGCAGGGTG
CACATGGTGATGCAGTTATTCTGGGTATGAGCAGCCTGGAACAGCTGGAACA
AAATCTGGCAGCAGCAGAAGAAGGTCCGCTGGAACCGGCAGTTGTTGATGC
CTTTAATCAGGCATGGCATCTGGTTACCCATGAATGTCCGAATTATTTCCGTTA
A

>AKR1C6 (**Uniprot: P70694**)
ATGGATTCAAAACAACAAACAGTAAGGCTAAGTGATGGGCACTTTATACCGA
TTCTGGGCTTTGGTACCTACGCGCCGCAAGAAGTGCCGAAGAGCAAAGCGA
CCGAAGCGACCAAGATTGCGATCGATGCGGGTTTTCGTCACATCGATAGCGC
GAGCATGTATCAGAACGAGAAGGAAGTTGGTCTGGCGATTCGTAGCAAGATT
GCGGACGGTACCGTGAAACGTGAGGACATCTTCTACACCAGCAAAGTGTGG
TGCACCTTTCACCGTCCGGAACTGGTTCGTGTGTGCCTGGAACAAAGCCTGA
AACAGCTGCAACTGGATTACGTGGACCTGTACCTGATCCACTTTCCGATGGC
GATGAAACCGGGCGAAAACTACCTGCCGAAGGACGAGAACGGTAAACTGAT

CTACGACGCGGTGGATATCTGCGATACCTGGGAAGCGATGGAGAAATGCAAG
GACGCGGGTCTGGCGAAGAGCATCGGTGTTAGCAACTTCAACCGTCGTCAG
CTGGAGAAGATCCTGAAAAAGCCGGGTCTGAAGTACAAACCGGTTTGCAAC
CAGGTTGAGTGCCACCCGTACCTGAACCAGGGTAAACTGCTGGATTTCTGCC
GTAGCAAAGACATCGTGCTGGTTGCGTACAGCGCGCTGGGTAGCCACCGTGA
GAAACAGTGGGTTGACCAAAGCAGCCCGGTTCTGCTGGATAACCCGGTTCTG
GGTAGCATGGCGAAGAAATATAACCGTACCCCGGCGCTGATTGCGCTGCGTT
ACCAGCTGCAACGTGGTGTTGTGGTTCTGGCGAAAAGCTTCAGCGAGAAGC
GTATCAAAGAGAACATGCAGGTTTTCGAGTTCCAACTGACCAGCGAGGACAT
GAAAGTGCTGGATGACCTGAACAAGAACATCCGTTACATCAGCGGTAGCAGC
TTCAAAGACCACCCGGACTTCCCGTTCTGGGACGAATACTAA
